# Up-Frameshift Suppressor 3 as a prognostic biomarker and correlated with immune infiltrates: A pan-cancer analysis

**DOI:** 10.1371/journal.pone.0273163

**Published:** 2022-10-04

**Authors:** Jianduo Xu, Hongqing Ma, Baoen Shan

**Affiliations:** 1 Department of General Surgery, Shijiazhuang People’s Hospital, Shijiazhuang, P. R. China; 2 Department of General Surgery, The Fourth Hospital of Hebei Medical University, Shijiazhuang, P. R. China; 3 Hebei Medical University, Shijiazhuang, P. R. China; 4 Research Centre, The Fourth Hospital of Hebei Medical University, Shijiazhuang, P. R. China; Ohio State University, UNITED STATES

## Abstract

**Background:**

The mRNA expression of protein Up-Frameshift Suppressor 3 Homolog B (UPF3B) differ in different tumors. However, the clinical relevance of UPF3B in cancer patients, such as with prognosis, tumor stage, and levels of tumor-infiltrating immune cells remain unclear.

**Methods:**

We performed bioinformatics analysis of UPF3B with The Cancer Genome Atlas (TCGA) database (https://xenabrowser.net) and TIMER2.0 (Tumor Immune Estimation Resource 2.0, http://timer.comp-genomics.org/). UPF3B expression in 33 cancers versus counterpart normal tissues was analyzed using TCGA pan-cancer data. The influence of UPF3B in long-term prognosis was evaluated using Kaplan–Meier method, and the associations between UPF3B transcription levels and immune-related gene expression, immune cell infiltration, tumor microenvironment (TME) score are analyzed by spearman correlation analysis. Enrichment analysis of UPF3B was conducted using the R package “clusterProfiler.”

**Results:**

The transcriptional level of UPF3B was dysregulated in the human pan-cancer dataset. A significant correlation was found between the expression of UPF3B and the pathological stage of Esophageal Carcinoma (ESCA), Kidney Chromophobe (KIHC), Liver Hepatocellular Carcinoma (LIHC), and Skin Cutaneous Melanoma (SKCM). Multiple cancer types with high transcriptional levels of UPF3B were associated with a significantly worse prognosis. The functions of expressed UPF3B gene are primarily related to ubiquitin mediated proteolysis, cell cycle, and mRNA surveillance pathway. Our results also show that immune cells infiltration and immunosuppressive markers such as CTLA-4, PD-1 and PD-L1 significantly correlate with UPF3B expression.

**Conclusions:**

In the present study, we synthetically explored the expression status and prognostic significance of UPF3B, and the relationship with clinic characters and immune microenvironment across cancers. Our results may provide novel insights for UPF3B as an immunotherapeutic target and valuable prognostic biomarker in various malignant tumor.

## Introduction

Cancer is rapidly increasing worldwide, leading to high morbidity and mortality [1]. On the basis of global data, lung, breast, and liver tumors are the major causes of high mortality rate worldwide [2]. Medical researchers had made great efforts to improve the efficacy of cancer treatment, such as precision tumor oncology [3], surgical technique [4]. In addition, immune checkpoint inhibition (ICIs) and targeted therapies [5] have revolutionized cancer treatment. However, heterogeneity within tumors presents a significant obstacle toward the survival rate for cancer patients [6]. Therefore, it becomes increasingly urgent for us to find diagnostic biomarkers and new treatment options for cancer.

A highly conserved RNA turnover pathway called the Nonsense-mediated RNA decay (NMD) that selectively degrades RNAs harbouring truncating mutations (frameshift, nonsense and splice site mutations) [7]. NMD play a role in the course of tumor progression through downregulating the expression of tumor suppressor genes [8]. Previous studies had found that Up-Frameshift Suppressor 3 Homolog B (UPF3B) is an core adaptor protein in this pathway and serves as an NMD amplifier [9]. However, the role of UPF3B in cancer genomes consisting of its relationship with tumorigenesis, early diagnosis, prognosis, and therapy wasn’t supported by studies. A thorough pan-cancer analysis isn’t also available.

In our analysis, we comprehensively investigated the expression of UPF3B and their prognostic value in pan-cancer through bioinformatic analysis. The more biological function and molecular mechanisms of UPF3B in tumor progression were also intensively explored.

## Materials and methods

### Genomic data collection

RNA-SEQ and clinical information of UPF3B from TCGA samples were downloaded from the Pan-Cancer TCGA project (contains 11069 samples across 33 types of cancer) using UCSC Xena tool (https://xena.ucsc.edu/). The protein expression data of 10 tumors were obtained from the UALCAN database (http://ualcan.path.uab.edu/).

### UPF3B gene expression analysis

The UPF3B expression in pan-cancer was evaluated using the downloaded data of 33 tumor tissues. The UPF3B differential expression analysis was performed in paired tumor and normal tissues of 1,362 patients in all. A total of 6,484 patients having the completed staging system were selected to analyze the UPF3B expression. Survival data were extracted for each of the patients including overall survival (OS), disease-specific survival (DSS), disease-free interval (DFS), and progression-free interval (PFI) information. The correlation between UPF3B expression and clinical outcomes in various cancer types was determined by generating Kaplan–Meier curve using the above data.

### Functional analysis of UPF3B and related genes

To identify UPF3B-related genes in LIHC, we calculated Pearson’s correlation coefficient using the TCGA-LIHC data. Based on the calculation results, the genes that were positively relevant to UPF3B were selected for functional enrichment analysis to unveil the underlying molecular mechanisms of UPF3B. To investigate the biological functions of UPF3B in cancers, we performed the Gene Ontology (GO), kyoto encyclopedia of genes and genomes (KEGG), and the gene set enrichment analyses (GSEA) by applying the R package “clusterProfiler”. The enriched gene sets in the above results that reached a nominal (NOM) significance level of P < 0.05 were considered significant.

### Immune infiltrates analysis

Single-Sample GSEA (ssGSEA) was utilized for quantifying the abundances of specific cell populations in a pooled population of cells, using gene expression data [10]. In this study, ssGSEA was used to calculate relative fractions for 24-immune cells in LIHC using TCGA RNA-seq dataset [11]. Different tumor subtypes are associated with the different tumor microenvironments (TME). A microenvironment comprehensive score [12] was used to reflect the effect of UPF3B on the tumor microenvironment in LIHC. We divided LIHC samples in the TCGA cohort into two groups (high UPF3B group and low UPF3B group) according to the median of the UPF3B expression to compare the pathway scores. Furthermore, survival impact and correlation between UPF3B and the myeloid-derived suppressor cells (MDSCs) were analyzed by the Tumor IMmune Estimation Resource (TIMER) online database (cistrome.shinyapps.io/timer).

### Statistical analysis

In the present study, we used R and corresponding R packages were utilized for statistical analysis. Student’s t-test was used to analyze differences in the analysis of continuous variables. Spearman’s correlation was adopted for the analysis of correlations. Kaplan–Meier survival analysis was performed to evaluate the prognostic value of UPF3B based on the TCGA cohort using the “survival” R package. Statistical significance was defined by a two-tailed *P* < 0.05 (*p < 0.05; **p < 0.01; ***p < 0.001; ****p < 0.0001).

### Tissue samples and reverse-transcription quantitative PCR (qPCR)

We retrospectively analyzed data from one cohort: Adult liver cancer patients admitted to The fourth hospital of Hebei medical university. Tissues samples were collected after hepatectomy from December 2020 to June 2021. Patients were excluded if they had received anticancer therapy, such as chemotherapy, radiotherapy, and other adjuvant treatments. A total of 14 liver cancer and paired non-cancerous tissues were taken and used for qRT-PCR. The human samples in our study were in compliance with the principles of Declaration of Helsinki concerning Principles for Medical Research containing Human Subjects. Experiment was approved by the ethics committee of The fourth hospital of Hebei medical university, China.

Written informed consent for participation was not required for this study in accordance with the national legislation and the institutional requirements. Bio-Rad CFX96 Real-Time System manager (C1000 Thermal Cycler) was used to detect the expression level of target genes using iTaq Universal SYBR Green, and GAPDH was used as the internal control. The primer sequences of UPF3B are FORWARD (5′-3′) CCTAAGGAGAAGCGAGTAACCC, REVERSE (5′-3′) CCTTGTTGCGATCCTGCTTATC. The primer sequences of Beta-actin are FORWARD (5′-3′) CATGTACGTTGCTATCCAGGC, REVERSE (5′-3′) CTCCTTAATGTCACGCACGAT.

## Results

### UPF3B is highly overexpressed in multiple tumors and correlated with advanced tumor stages

**Fig 1:**
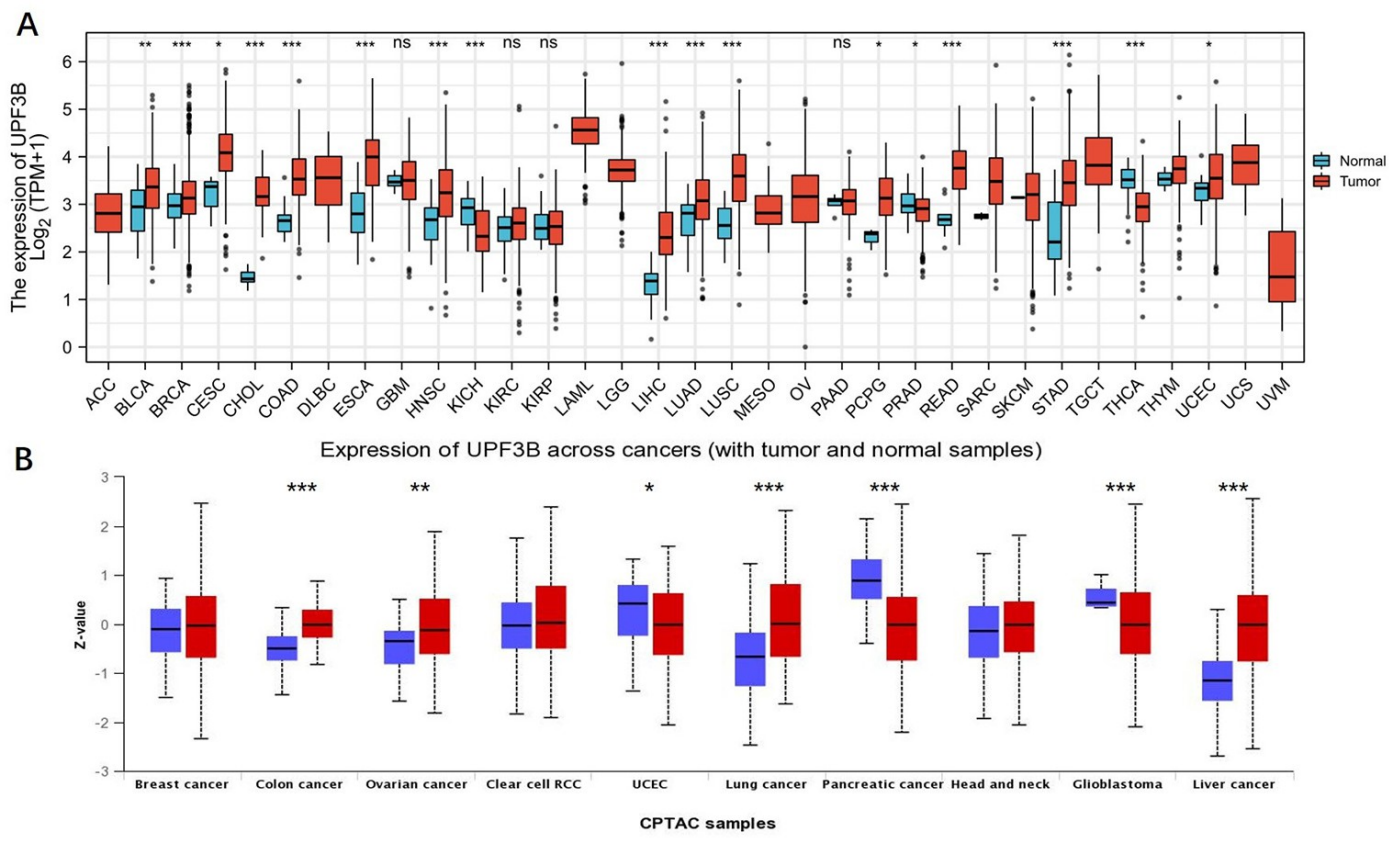
The transcription levels of UPF3B in human cancers. (A, B) The expression of UPF3B in different type of tumor tissues and normal tissues. Data was obtained via TCGA. (B)The protein levels of UPF3B in different types of cancers determined using UALCAN databases. *P < 0.05, **P < 0.01, ***P < 0.001.

**Fig 2:**
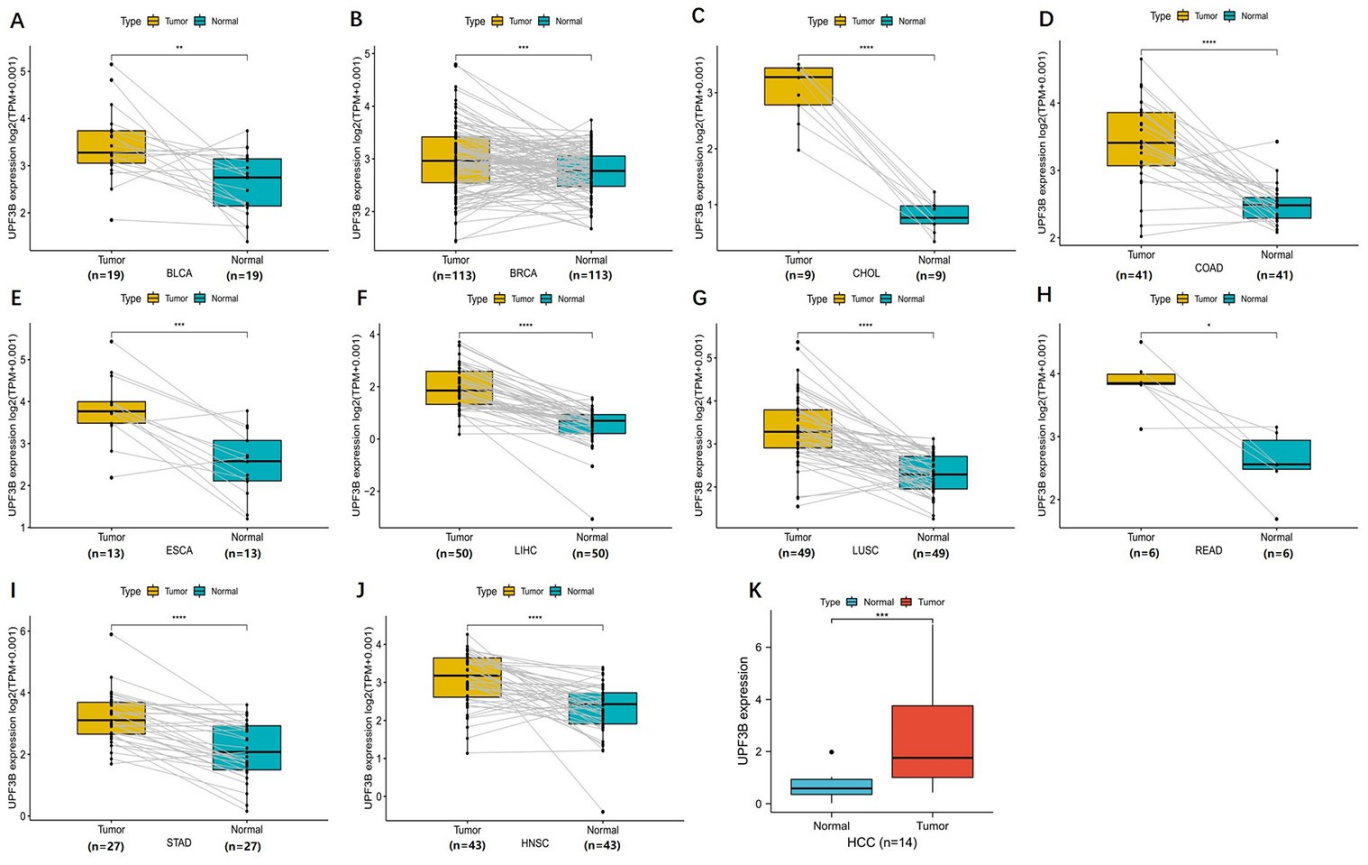
Paired expression analysis of UPF3B in pan-cancer and validate UPF3B expression in liver tumor samples. (A-J) UPF3B expression in indicated paired tumor and normal tissues in pan-cancer data of TCGA. Gray lines connect paired tissues. (K) qRT-PCR results showed the expression of UPF3B in liver cancer tissues. Data was obtained via The Fourth Hospital of Hebei Medical University. *p < 0.05, **p < 0.01, ***p < 0.001, ****p < 0.0001.

Based on the RNA sequencing data in TCGA, we first evaluated the expression profiles of UPF3B across various types of cancers (including those cancers without normal tissues for comparison). The analysis revealed that UPF3B expression was higher in 13 tumors, including Bladder Urothelial Carcinoma (BLCA), Breast Invasive Carcinoma (BRCA), Squamous cell carcinoma of the cervix (CESC), Cholangiocarcinoma (CHOL), Head and Neck Squamous Cell Carcinoma (HNSC), ESCA, Colon Adenocarcinoma (COAD), LIHC, Lung Adenocarcinoma (LUAD), Lung Squamous Cell Carcinoma (LUSC), Paraganglioma (PCPG) and Pheochromocytoma, Rectum Adenocarcinoma (READ), Stomach Adenocarcinoma (STAD). Conversely, UPF3B is expressed at a low level in Kidney Chromophobe (KICH), Prostate Adenocarcinoma (PRAD), Thyroid Carcinoma (THCA). However, there was no significant UPF3B expression difference in Glioblastoma Multiforme (GBM), Kidney Renal Clear Cell carcinoma (KIRC), Kidney Renal Papillary Cell Carcinoma (KIRP), Pancreatic Adenocarcinoma (PAAD), SKCM, Sarcoma (SARC), Thymoma (THYM) and Uterine Corpus Endometrial Carcinoma (UCEC) (Fig 1A). Proteomic data of UPF3B in cancer was got from UALCAN (contains 10 cancer protein expression data). As shown in Fig 1B, the proteins level of UPF3B was significantly increased in different types of cancers, including Colon cancer, Ovarian cancer, lung cancer, Liver Cancer. In contrast, UPF3B protein expression was markedly decreased in UCEC, pancreatic cancer, and glioblastoma. For paired samples of tumors and normal tissues, UPF3B was overexpressed in tumor tissues of BRCA, BLCA, ESCA, CHOL, LIHC, COAD, LUSC, STAD, READ and HNSC (Fig 2A–2J).

**Fig 3:**
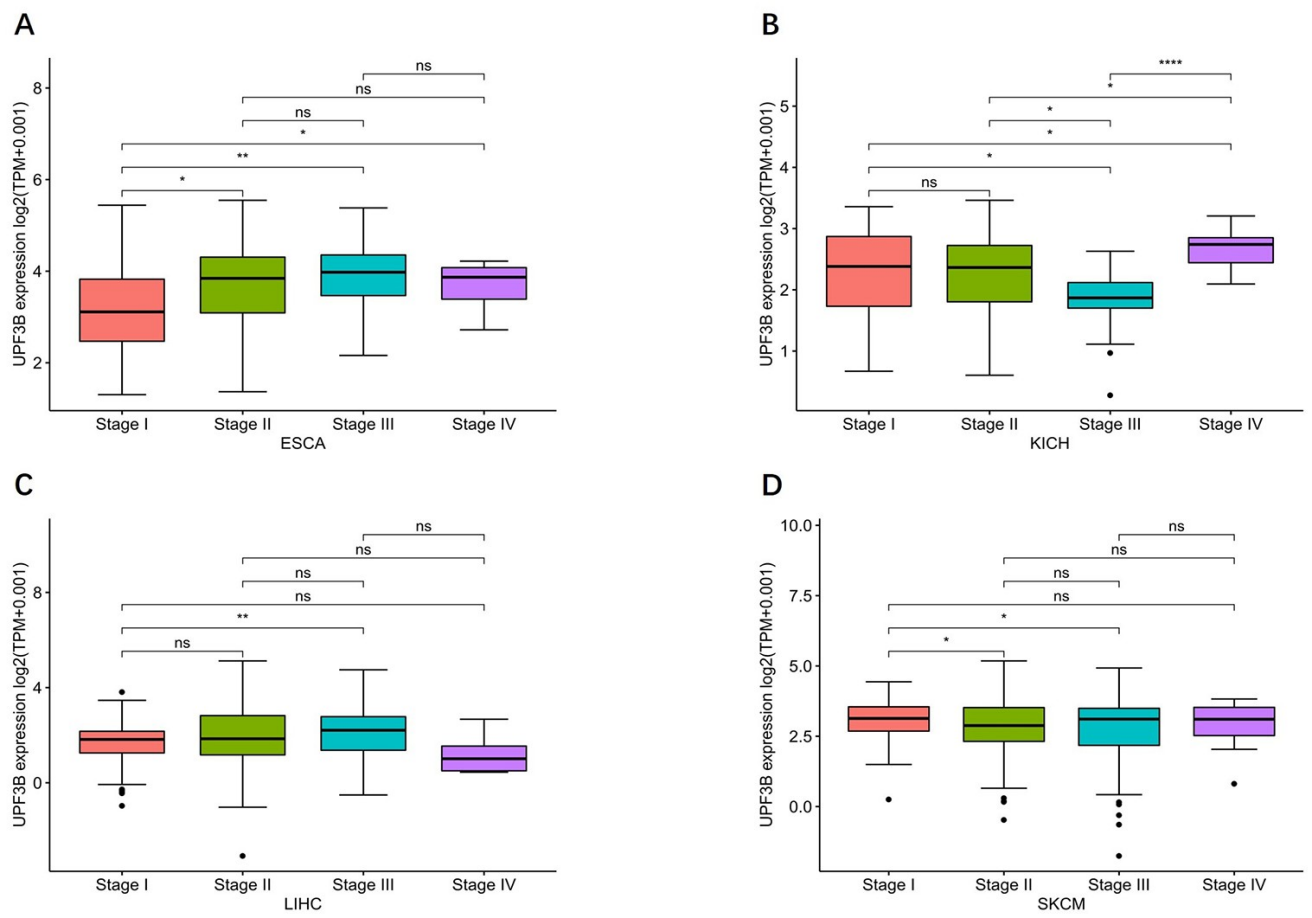
Association between UPF3B expression and tumor stages. (A) Esophageal carcinoma (ESCA). (B) kidney chromophobe (KICH). (C)liver hepatocellular carcinoma (LIHC). (D) skin cutaneous melanoma (SKCM). Data was obtained via TCGA. *p < 0.05, **p < 0.01, ***p < 0.001, ****p < 0.0001.

Moreover, we performed a qRT-PCR to validate the expression of UPF3B using 13 paired samples from liver cancer patients. The results revealed that HILPDA was highly expressed in liver cancer tissues (Fig 2K). We proceeded to analyzed the correlation of UPF3B expression with tumor stage and found that UPF3B expression significantly correlated with tumor stage in ESCA and LIHC. In KICH and SKCM, the UPF3B expression elevated slightly but significantly with higher tumor stage. As shown in Fig 3A–3D, UPF3B expression increased from stage I to stage III in ESCA, SKCM, and LIHC. Moreover. UPF3B expressed Markedly higher at stage IV than other stages in KIHC.

### Relationship between UPF3B expression and cancer survival

**Fig 4:**
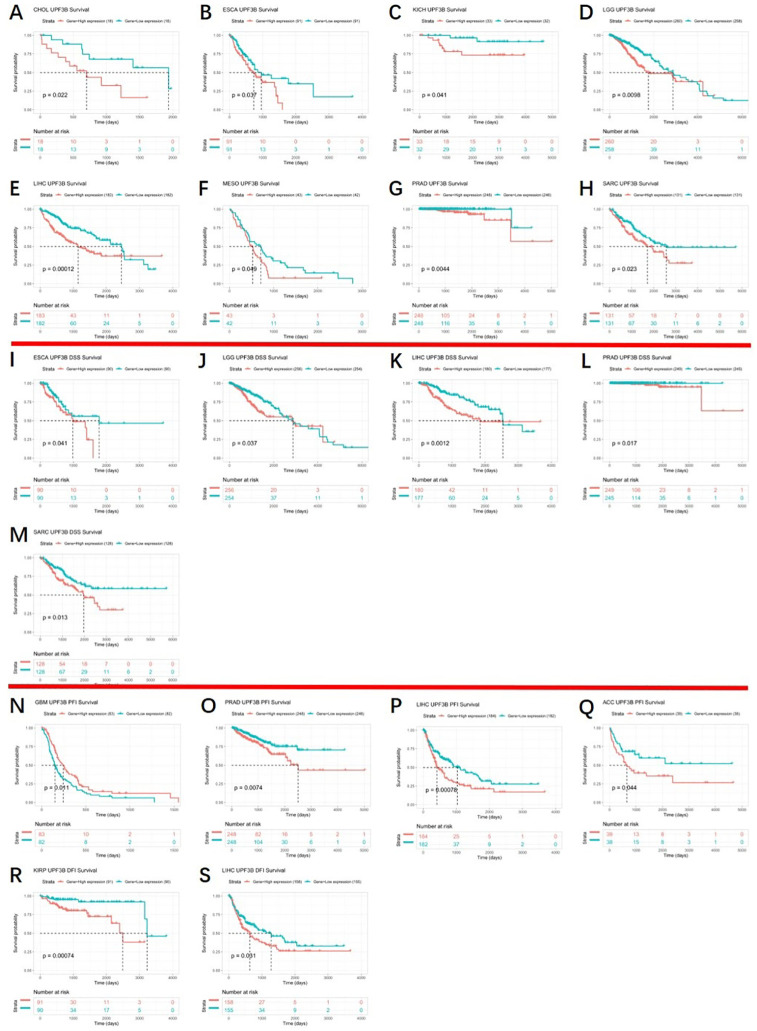
Kaplan–Meier analysis of patient prognosis in 33 TCGA tumor types, group division was based on the median of UPF3B expression. (A-H) Association between UPF3B expression and OS of cancer patients. (I-M) Correlations of UPF3B expression with DSS of patients. (N-Q) Correlations of UPF3B expression with PFI of patients. (R, S) Correlations of UPF3B expression with DFI of patients. Data was obtained via TCGA. *p < 0.05, **p < 0.01, ***p < 0.001, ****p < 0.0001.

Because we found that UPF3B was aberrantly expressed in several cancers and correlated with tumor progression, the correlation analysis of the UPF3B expression and clinical outcomes across different cancers was conducted in the TCGA cohort. Fig 4 shows the KM curves of the association between UPF3B expression and the survival data of cancer patients, including overall survival (OS), Disease Free Survival (DSS), progression Free interval (PFI), and disease-free interval (DFS). We found that higher expression of UPF3B group was significantly associated with worse OS than its low expression in CHOL (*p =* 0.022), ESCA (*p =* 0.037), KICH (*p =* 0.041), LGG (*p =* 0.0098), LIHC (*p =* 0.00012), MESO (*p =* 0.049), PRAD (*p =* 0.0044) and SARC (*p =* 0.0023) (Fig 4A–4H). In ESCA (*p =* 0.041), LGG (*p =* 0.037), LIHC (0.0012), PRAD (*p =* 0.017) and SARC (*p =* 0.013), the DSS of patients with high expression UPF3B to be shorter (Fig 4I–4M). Similarly, high expression of UPF3B was significantly associated with a decreased PFI in PRAD (*p =* 0.0074), LIHC (*p =* 0.00078) and ACC (*p =* 0.044) (Fig 4O–4Q). But, the PFI was reduced in the low UPF3B expression group at GBM (*p =* 0.011) (Fig 4N). In addition, we observed that patients with high UPF3B expression had worse DFI than those with a low expression of UPF3B in KIRP (*p =* 0.00074) and LIHC (*p =* 0.0031) (Fig 4R and 4S). The remainder of differences were not significant (S1 File). These findings clearly demonstrate that UPF3B can be used as a biomarker to determine the prognosis of various cancers.

### Gene sets enriched in UPF3B expression phenotype

**Fig 5:**
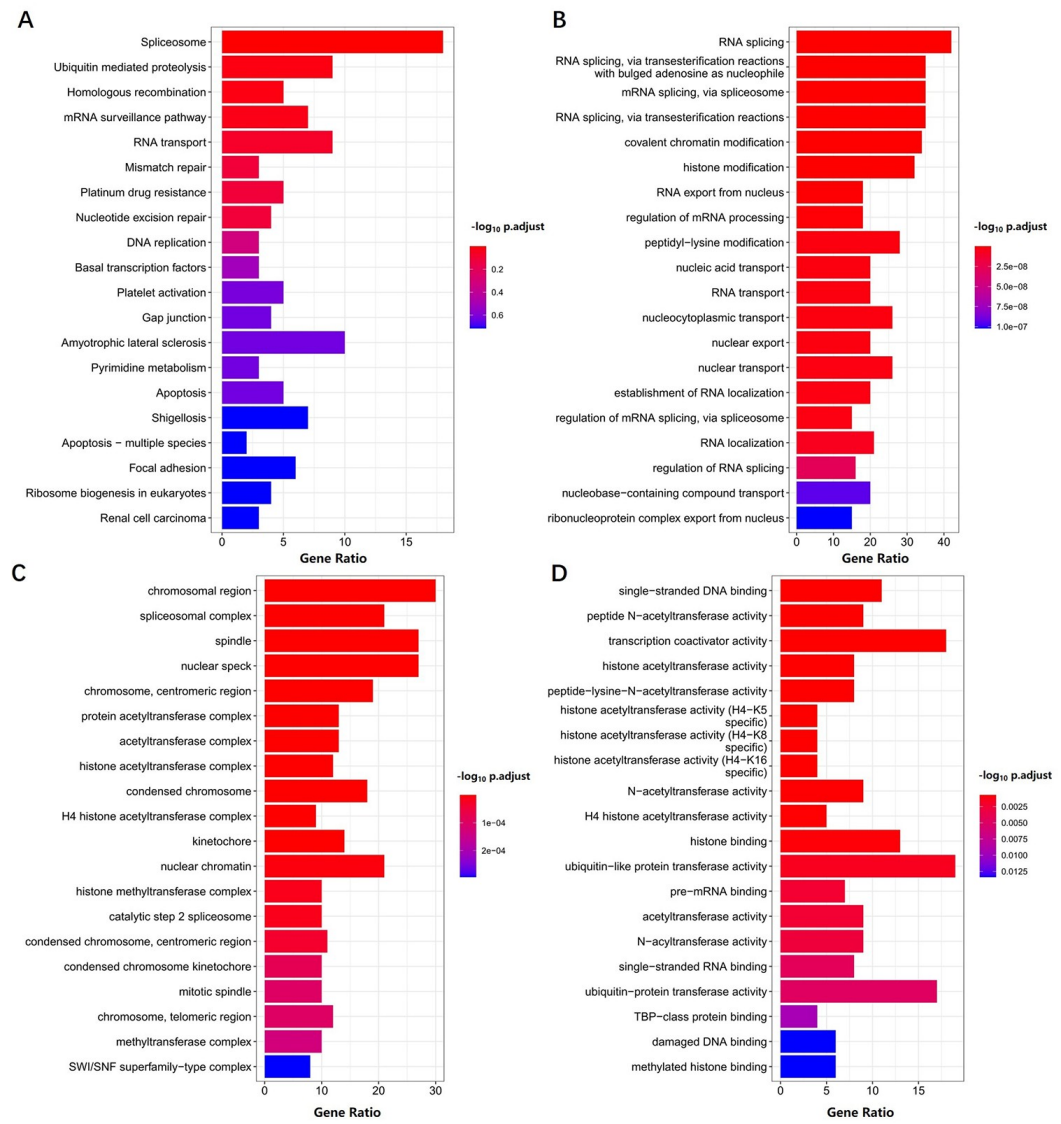
Functional enrichment analyses of differentially expressed genes (DEGs) in high- and low- UPF3B expression LIHC samples. KEGG (A)and Gene Ontology (GO) analyses, including biological process (B), cellular component(C) and molecular function (D) were performed by clusterProfiler R package. Data was obtained via TCGA. P value < 0.05 was considered statistically significant.

**Fig 6:**
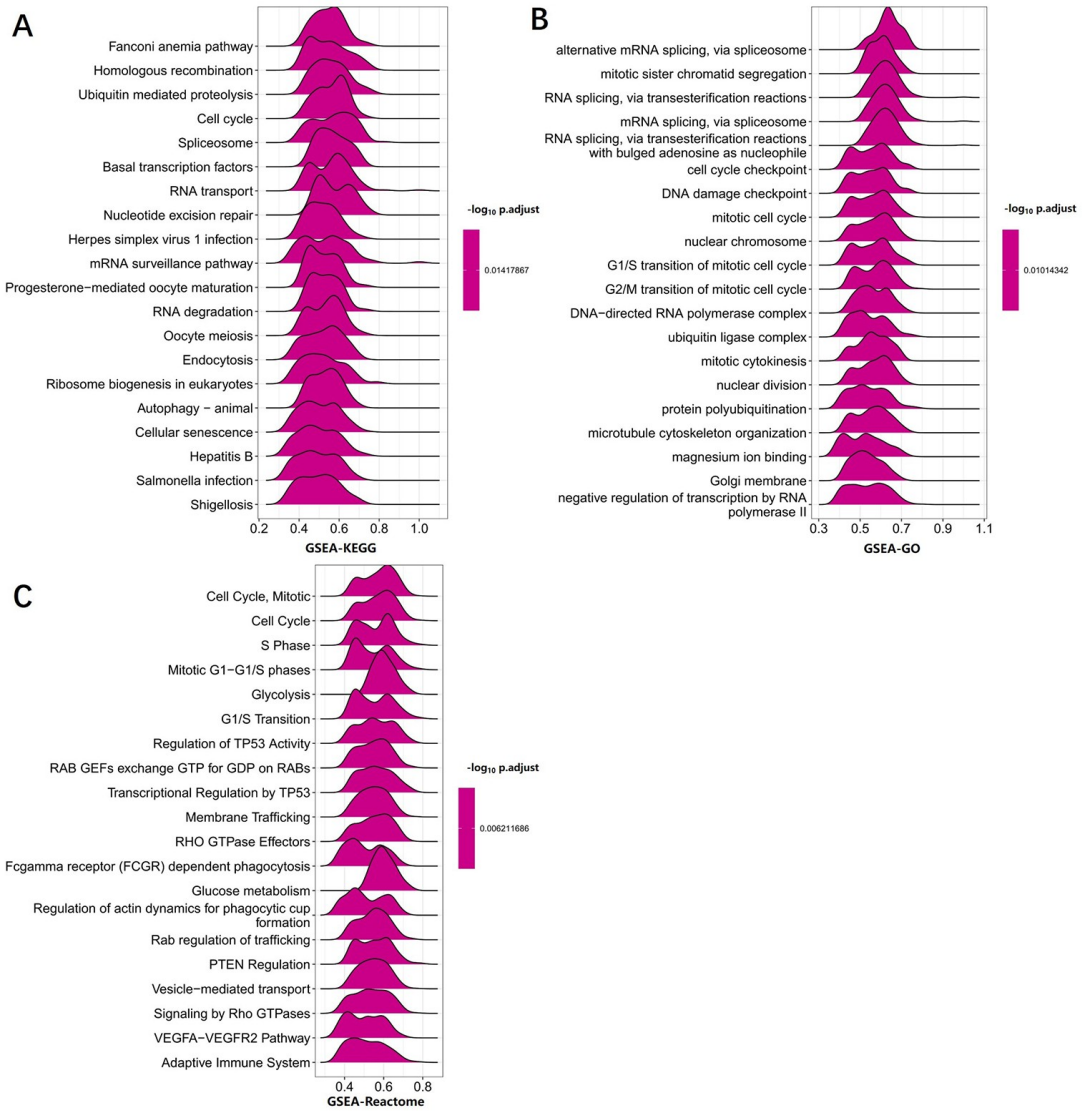
GSEA analysis revealed the pathway enriched in UPF3B high and low expression phenotype. (A) GSEA analysis in KEGG gene sets. (B) GSEA analysis in GO gene sets. (C) GSEA analysis in ReactomE gene sets. Data was obtained via TCGA-LIHC. Only gene sets with NOM p < 0.05 and FDR q < 0.06 were considered significant.

Because UPF3B is abnormally expressed in LIHC and is associated with the prognosis of LIHC patients, we further analyzed its potential molecular mechanism in LIHC. In order to examine the UPF3B-associated biological functions in LIHC, we performed gene enrichment analysis on all the DEGs identified between sorted UPF3B-high and UPF3B-low subpopulations. Gene Ontology (GO) enrichment analysis shows that several RNA splicing-related pathways were enriched, including RNA splicing, via transesterification reactions with bulged adenosine as a nucleophile (GO:0000377), mRNA splicing, via spliceosome (GO:0000398) and RNA splicing, via transesterification reactions (GO:0000375). These DEGs were highly enriched in cellular components related to subnuclear structure such as spliceosomal complex (GO:0005681), nuclear speck (GO:0016607). UPF3B was also significantly enriched for multiple pathways in the Kyoto Encyclopedia of Genes and Genomes (KEGG), including Spliceosome (hsa03040), Ubiquitin mediated proteolysis (hsa04120) and mRNA surveillance pathway (hsa03015) (Fig 5A–5D). We further conducted a GSEA analysis of UPF3B using LIHC-TCGA data. The results of GSEA showed that RNA degradation and cell cycle pathway in KEGG and mRNA splicing, DNA damage checkpoint in GO were significantly enriched (Fig 6A and 6B). Moreover, cancer pathways were also significantly enrichment in Reactome: regulation of TP53 activity, Rab regulation of transport, and PTEN regulation (Fig 6C). Misregulation of these pathways and functions is associated with many pathologies, including malignancies.

### Correlation between UPF3B and immunological infiltrate in tumor

The immune-related mRNA can predict the composition of the immunizing cells in tumor tissues [11], and the tumor-infiltrating lymphocytes also harbor the role of a biomarker for immunotherapy and prognosis [13]. We further investigated the effect of UPF3B on the tumor microenvironment (TME).

**Fig 7:**
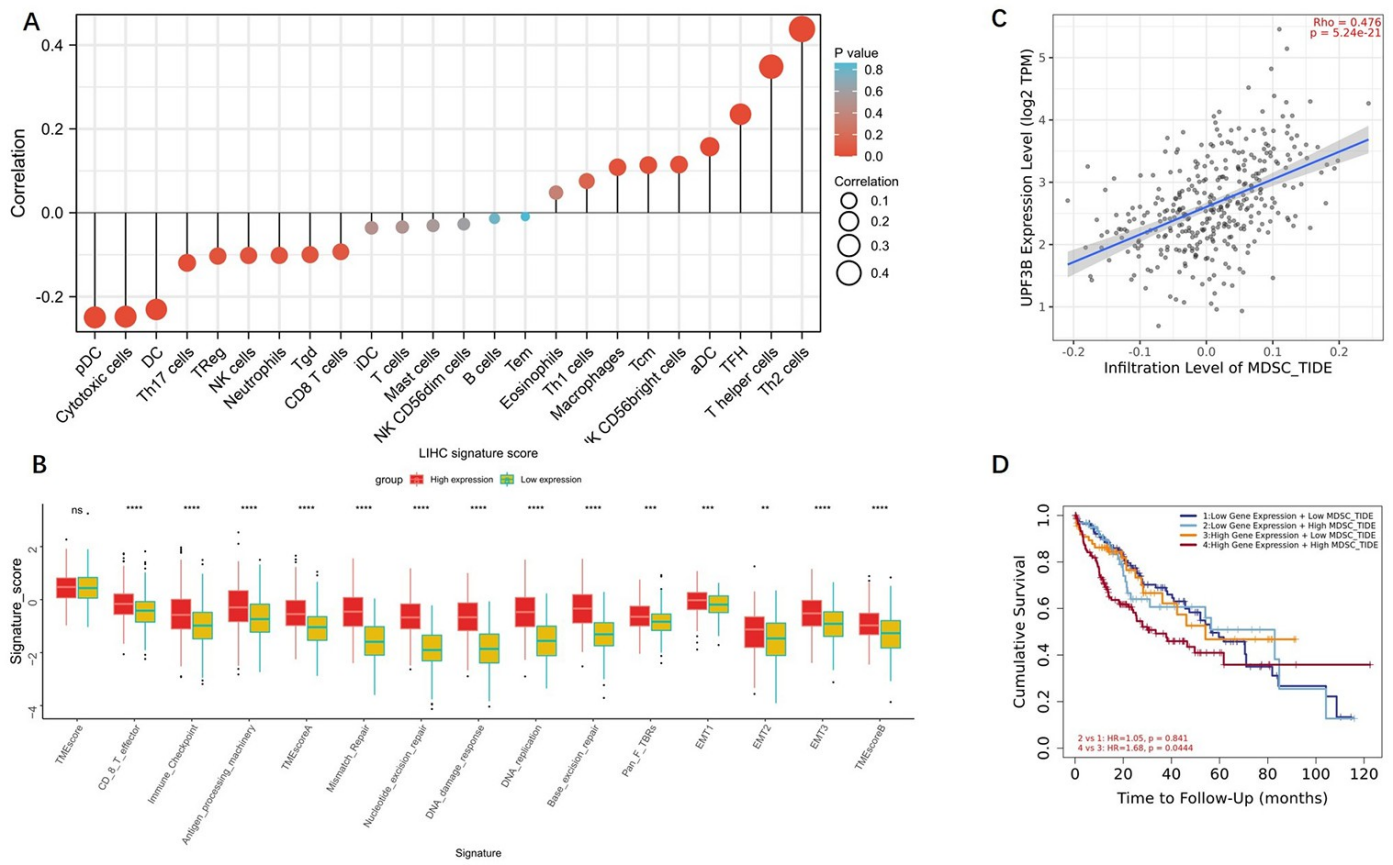
Correlation analysis of UPF3B level and immune microenvironment in LIHC. (A) UPF3B expression in LIHC tissues positive correlates with seven immune cell types. (B) Multiple TME scores in up-regulated UPF3B tumor tissues was significantly higher than down-regulated UPF3B tumor tissues. (D) KM curves according UPF3B expression and MDSC Infiltrating level in LIHC. *p < 0.05, **p < 0.01, ***p < 0.001, ****p < 0.0001.

Using the ssGSEA method, we calculated the percent of 24 leukocyte cells of liver cancer samples in TCGA. As shown in Fig 7A, UPF3B expression was positively correlated with seven immune cells in LIHC, including Macrophages, Tcm, NK CD56bright cells, aDC, TFH, T helper cells and Th2 cells. Meanwhile, UPF3B expression are negatively associated with pDC, Cytotoxic cells, DC, Th17 cells and Treg in liver tumor tissues (Fig 7B). To investigate the clinical predictive value of UPF3B for cancer immunotherapy, we calculated 15 TME scores which have a close association with immunotherapeutic outcomes [12]. The results summarized in Fig 7B show that the high UPF3B expressing liver cancer patients had higher TME scores as compared to the subset of patients showing lower UPF3B expression (TMEscoreA, B, Immune Checkpoint and 12 related pathways score).

Moreover, we also found a positive correlation between the UPF3B expression at LIHC and the MDSCs infiltration is remarkable (*p*<0.05) (Fig 7C). In addition, we compared survival between the high and low MDSCs groups both with overexpression UPF3B gene. As shown in Fig 7D, Prognosis is worse in patients with high levels of MDSCs and UPF3B. MDSCs play an important role in T cell dysfunction and hepatocarcinogenesis, may promote immune escape of liver tumor [14]. Overall, the results demonstrated that UPF3B had a strong relationship with immune cell infiltration and TME in LIHC.

### Relationships between UPF3B expression and immunoinhibitors, immunostimulators, and MHC-related genes in pan-cancer

**Fig 8:**
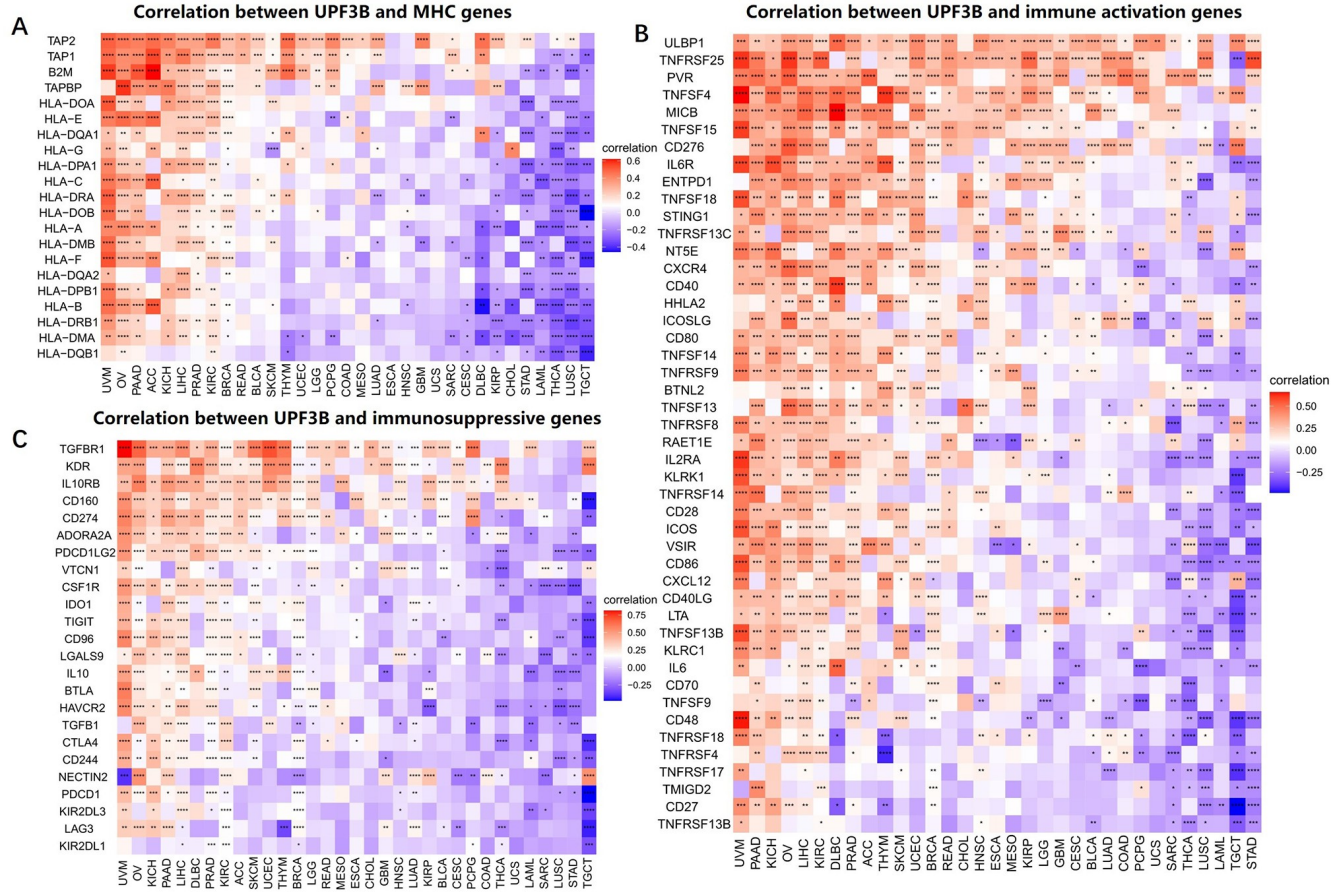
Co-expression of UPF3B and immune-related genes. (A) Correlation between UPF3B mRNA expression and MHC molecules. (B) Correlation between UPF3B mRNA expression and immune activation genes. (C) Correlation between UPF3B mRNA expression and immunosuppressive genes. *p < 0.05, **p < 0.01, ***p < 0.001, ****p < 0.0001.

Growing experimental evidences supported that the tumor immune microenvironment has a vital role in pro-tumorigenic and immunosuppressive (22, 23). Immune-related genes which encoded MHC, immune activation and immunosuppressive protein are important in promoting the immune system to respond to tumor-specific antigens [15, 16]. Hence, we adopted co-expression analyses to inference the relationships between immune-related genes and UPF3B expression in 33 tumors. There was a significant positively correlation between the UPF3B expression and the expression of different MHC-related genes in UVM, OV, PAAD, ACC, LIHC and PRAD. In contrast, UPF3B expression was negatively and strongly correlated with most MHC-related genes in STAD, LAML, THCA, LUSC and TGCT. (Fig 8A). Moreover, we found that the expression of UPF3B was significantly positive associated with immunostimulators in UVM, PAAD, KIHC, OV, LIHC and KIRC (Fig 8B). Additionally, UPF3B expression was positively correlated with most immunoinhibitors, including TGFBR1, IL10RB, CD160, CD274, TIGIT and PDCD1 in UVM, OV, KIHC, and LIHC, but negatively associated with the expression of majority genes in STAD and Testicular Germ Cell Tumors (TGCT) (Fig 8C). Hence, these results confirm our speculation that UPF3B expression in LIHC, PAAD, and UVM correlate with immunosuppressive status, which can help explain the differences in patient survival.

## Discussion

UPF3B gene encodes a protein involved in the nonsense-mediated mRNA decay (NMD). The NMD pathway is an RNA surveillance mechanism that can target diverse RNAs for degradation [17]. Since tumor suppressor genes mutated in cancer were degraded through NMD, it is commonly thought to protect against tumorigenesis [18]. The mutation of UPF3B causes multiple psychiatric illnesses, such as intellectual disability (ID) and schizophrenia (SCZ) [19]. Studies also suggest the UPF3B can be used as factors of gene models to predict tumor progression in various types of tumors, including esophageal cancer, liver cancer [20, 21]. However, its potential action in cancer has not been extensively studied. Therefore, it will be important to clarify the potential of modulating UPF3B gene for therapeutic purposes in this setting.

In the present study, we investigated the mRNA levels of UPF3B and asked whether UPF3B abnormal expression was prognostic for patient survival each tumor using TCGA pan-cancer data. According to our results, UPF3B was overexpressed in BLCA, BRCA, CESC, COAD, CHOL, ESCA, LIHC, HNSC, LUAD, LUSC, PCPG, READ and STAD while lower expression was observed in KICH, PRAD and THCA in TCGA. The protein levels of UPF3B also corresponded to mRNA expression in colon cancer, lung cancer and liver cancer but in opposite direction in pancreatic cancer and glioblastoma. The difference in UPF3B mRNA and protein levels in different tumor types may reflect the distinct underlying functions and mechanisms. We further found that upregulated of UPF3B generally predicts poor prognosis in tumors with high UPF3B expression, such as CHOL, ESCA, KICH, LGG, LIHC, MESO, PRAD and SARC. These results show that UPF3B can be further explored as a prognostic biomarker for tumor patients. Previous research had reported that UPF3B functions are critical for neuronal differentiation of neural stem cells [22]. Overexpressed UPF3B can be regarded as a DNA-damage marker in oligodendrocyte precursor cells, and related to a statue of replication stress prior to Gliomagenesis [19]. In this study, some specific molecular mechanisms were identified in the enrichment analysis part for LIHC data. Our enrichment analyses indicated that the strongly expressed UPF3B gene can potentially impact multiple critical links in RNA splicing process in LIHC, like transesterification reactions, regulation of spliceosome. The splicing patterns of mRNA were changed due to dysregulation or alterations involving splicing factors in t lymphoblastic leukemia cells, the efficient splicing of RNA can produce a sufficient amount of protein to adapt synthetic pressure in tumor cells [23]. Lu et al. reported that indisulam (degradation agent for splicing factor RBM39) can significantly inhibit in vivo growth of tumor cells in mice [24]. We also found the mitosis, G1/S transition and so on that involved in cell cycle processes were greatly affected in liver cancer patients with high UPF3B. There are also anomalies in regulation TP53 activity in these subset LIHC. This is probably because the aberrant p53 function in carcinoma allows tumor cells to enter mitosis despite showing DNA damage [25].

The tumor microenvironment, especially composition of immune cell infiltrates, is an important component of tumor biology. The generated data from RNA-seq goes beyond genomic descriptive information to include data on clinicopathological significance, such as biomarkers for diagnostic, prognostic and predicting therapeutic effect (responsive vs resistant) [26]. TMEscore is one of the quantitative biomarkers for predicting the patients’ prognostication after receiving the immune checkpoint inhibitors [27]. Our results indicated that multiple TMEscores were significantly positively related to UPF3B expression in the liver tumor.

A balanced T helper 1 cell (Th1)/ T helper 2 cells (Th2) response is preferable because it can suppress the harmful immune response in the human body [28]. In this study, with UPF3B up-regulated, the elevation of Th2 was more obvious than Th1 in LIHC, another study also come up with key genes aberrant expression that can shift the balance from pro‐inflammatory Th1 towards the immunosuppressive Th2 subtype in the TME. MDSCs are immature myeloid cells and identified with strong immunosuppressive functions [29]. Notably, we demonstrate that MDSC infiltration was significantly increased and survival was shorter in LIHC patients with high UPF3B expression.

Depletion in MHC class I molecules is a character for tumors with immune desert subtype [30]. It is well established that relieved immune suppression of immune cells such as exhausted CD8 T cells by immune checkpoints such as PD1, PD-L1 and restore their antitumor immunity [31]. Taking this further, we founded that UPF3B exhibited strong negative or positive association with MHC, immunosuppressive and immune activation genes in other tumors, this can be taken as proof of UPF3B involvement in formation and maintenance of complex TME. It is noteworthy that the expression of UPF3B was greatly associated with most immunoinhibitors, including PD-1, PD-L1, and CTLA4, in UVM, OV, KICH, and LIHC. Collectively, these results all indicate that expression of UPF3B is closely related to immune infiltration of tumor cells, affects patient prognosis in multiple tumors, providing a potential target for immunotherapy.

In summary, we first investigated the expression of UPF3B in pan cancer-TCGA and the liver cancer tissues collected from our own hospital to validate our analysis, the results showed that it differentially expressed between tumor and normal tissues. The results also revealed correlations of UPF3B expression with diverse cancer patients’ clinical prognoses. Our findings suggest that UPF3B can be proposed as an independent marker of prognosis of multiple tumors, the mRNA expression of UPF3B will bring different prognostic outcomes. Despite these data collected from Reliable quality datasets, several limitations should be acknowledged. First, several cancers lack a control group, further investigating the expression and function of UPF3B using a large sample size is necessary. Second, the prognostic value and some key signaling pathways in diverse tumors needed of in vitro or in vivo experiments to verify these findings. Third, more in vivo and in vitro experiments should be done to test the antitumor activity by targeting UPF3B and the role in regulating tumor immune microenvironment.

## Conclusion

On the whole, UPF3B expression was associated with immune cell infiltration in LIHC. These findings are beneficial to clarify the role of UPF3B in tumor progression and can provide a reference for the implemented of high precise and personalized anti-tumor immunotherapy in the future.

## Supporting information

S1 File (ZIP)
