# Supplementary figures and images for: Up-Frameshift Suppressor 3 as a prognostic biomarker and correlated with immune infiltrates: A pan-cancer analysis
Source: PLoS One. 2022 Oct 4;17(10):e0273163. doi: 10.1371/journal.pone.0273163 (PMC9531787; doi:10.1371/journal.pone.0273163)

# ACC UPF3B Survival

Strata + Gene=High expression (39) + Gene=Low expression (38)

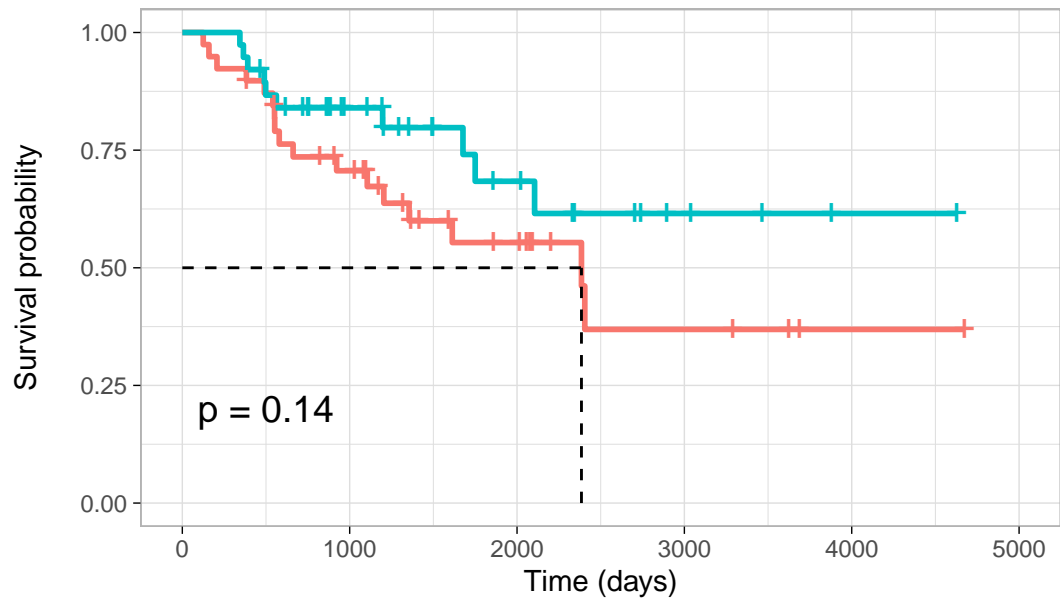

## Number at risk

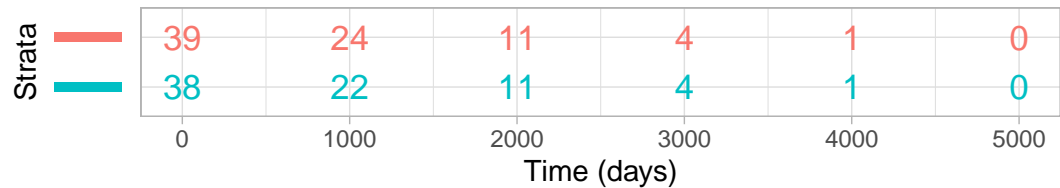

Supplement: S1 File — (ZIP) [file pone.0273163.s001.zip › Supplementary 1/5 UPF3B ACC ╔·┤μ╖╓╬÷.pdf]

# BLCA UPF3B Survival

Strata + Gene=High expression (203) + Gene=Low expression (202)

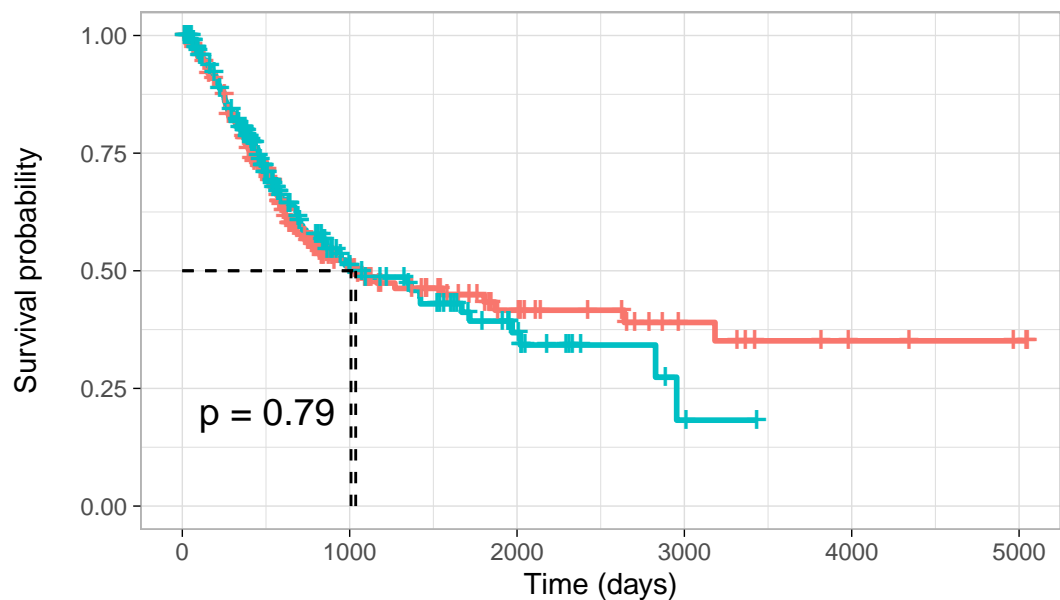

## Number at risk

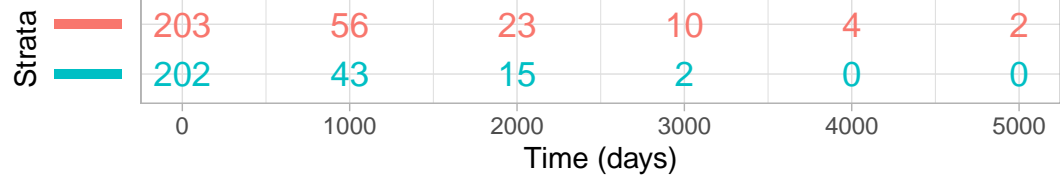

Supplement: S1 File — (ZIP) [file pone.0273163.s001.zip › Supplementary 1/5 UPF3B BLCA ╔·┤μ╖╓╬÷.pdf]

# BRCA UPF3B Survival

Strata    +    Gene=High expression (544)    +    Gene=Low expression (540)

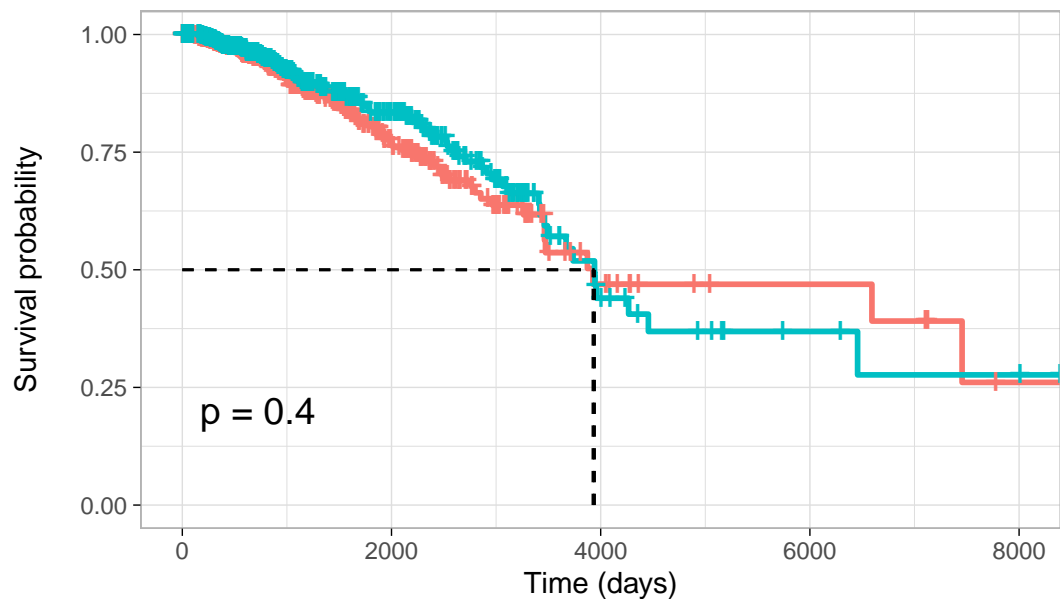

## Number at risk

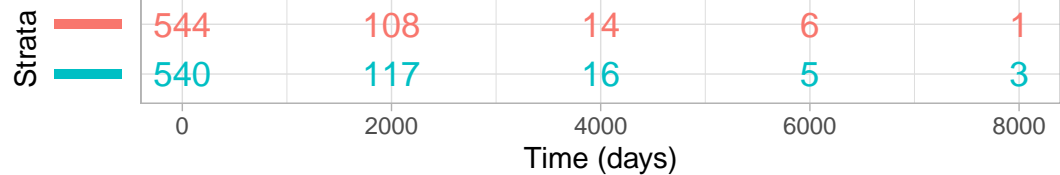

Supplement: S1 File — (ZIP) [file pone.0273163.s001.zip › Supplementary 1/5 UPF3B BRCA ╔·┤μ╖╓╬÷.pdf]

# CESC UPF3B Survival

Strata + Gene=High expression (147) + Gene=Low expression (146)

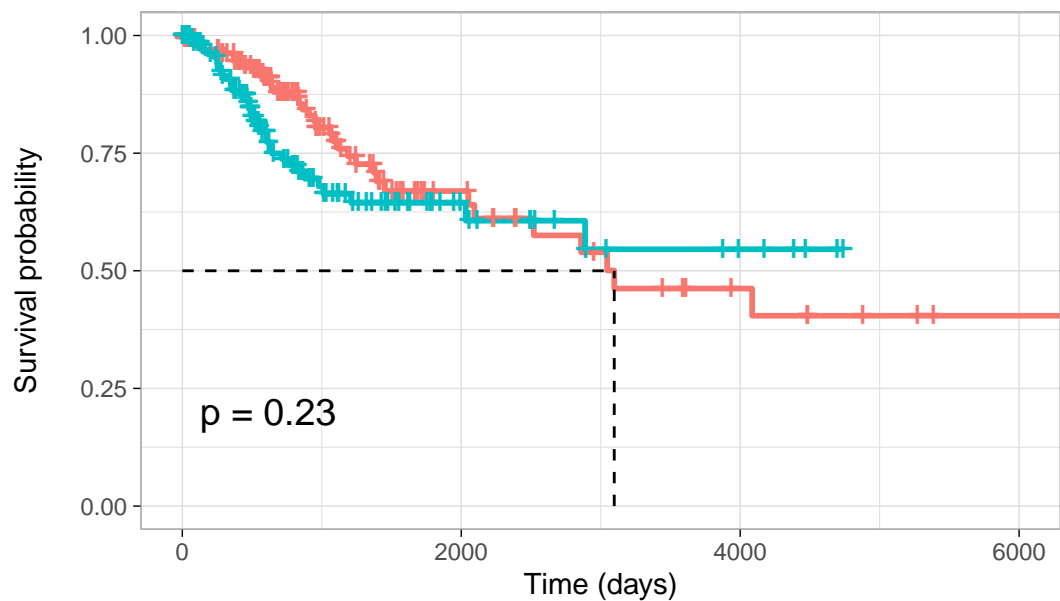

## Number at risk

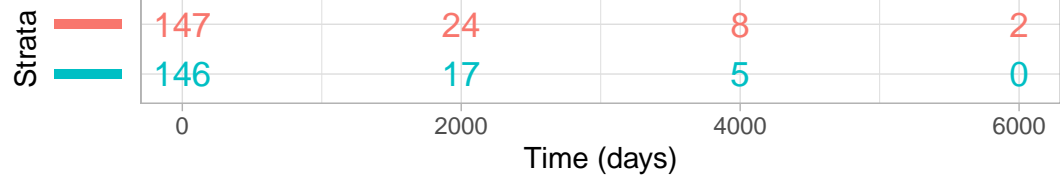

Supplement: S1 File — (ZIP) [file pone.0273163.s001.zip › Supplementary 1/5 UPF3B CESC ╔·┤μ╖╓╬÷.pdf]

# CHOL UPF3B Survival

Strata + Gene=High expression (18) + Gene=Low expression (18)

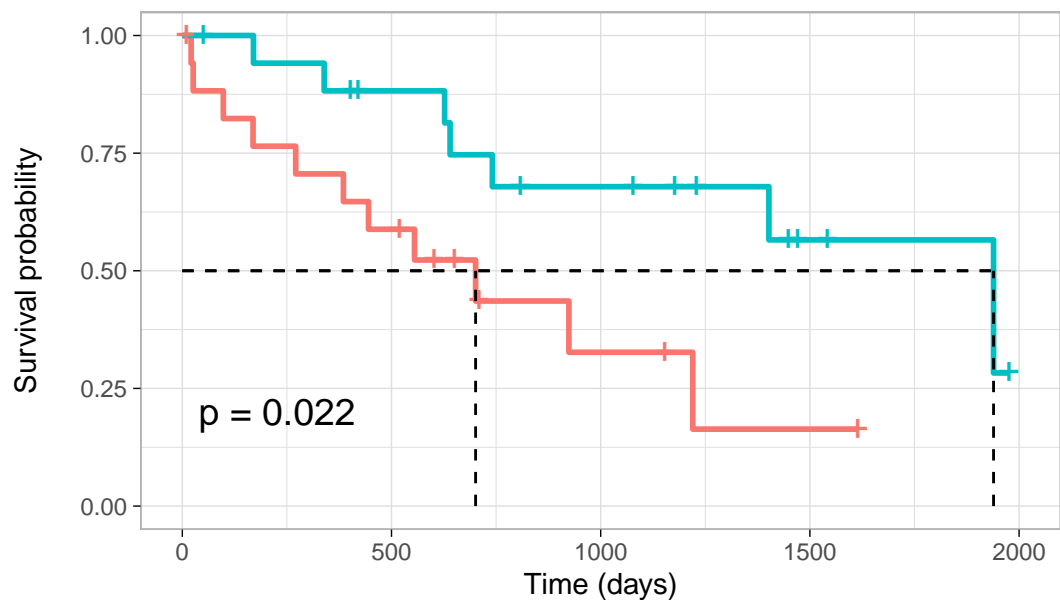

## Number at risk

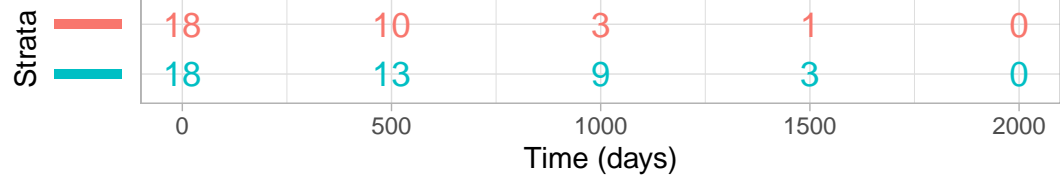

Supplement: S1 File — (ZIP) [file pone.0273163.s001.zip › Supplementary 1/5 UPF3B CHOL ╔·┤μ╖╓╬÷.pdf]

# COAD UPF3B Survival

Strata + Gene=High expression (143) + Gene=Low expression (141)

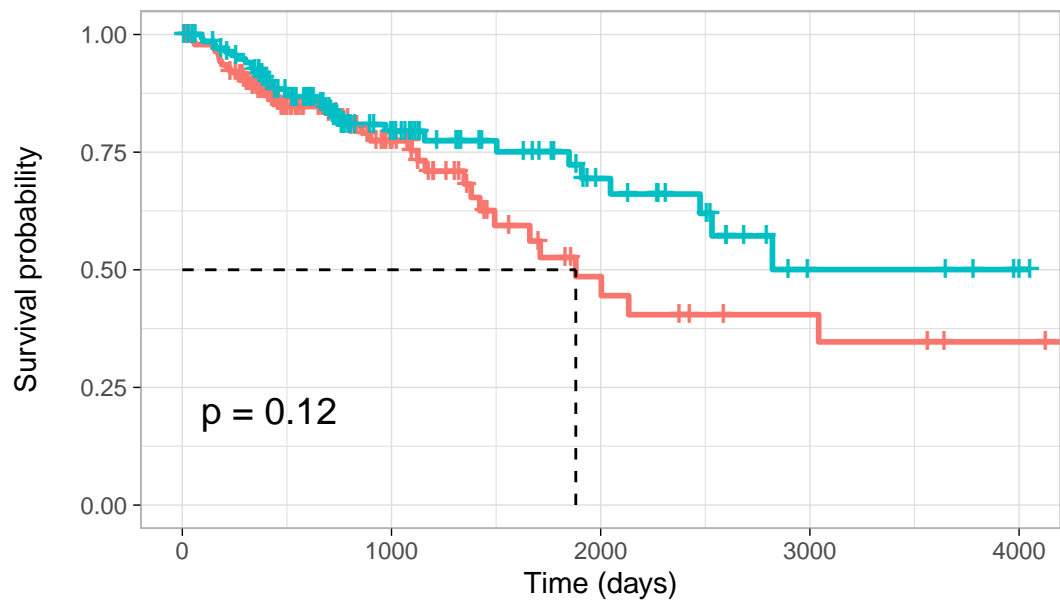

## Number at risk

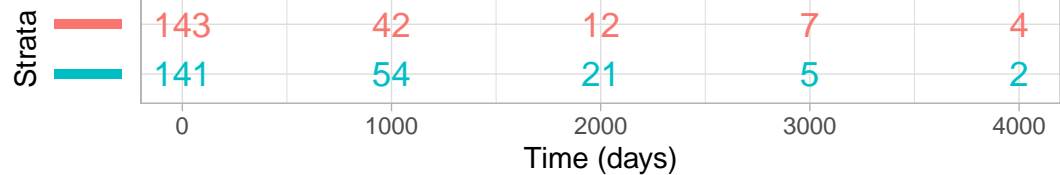

Supplement: S1 File — (ZIP) [file pone.0273163.s001.zip › Supplementary 1/5 UPF3B COAD ╔·┤μ╖╓╬÷.pdf]

# DLBC UPF3B Survival

Strata + Gene=High expression (23) + Gene=Low expression (23)

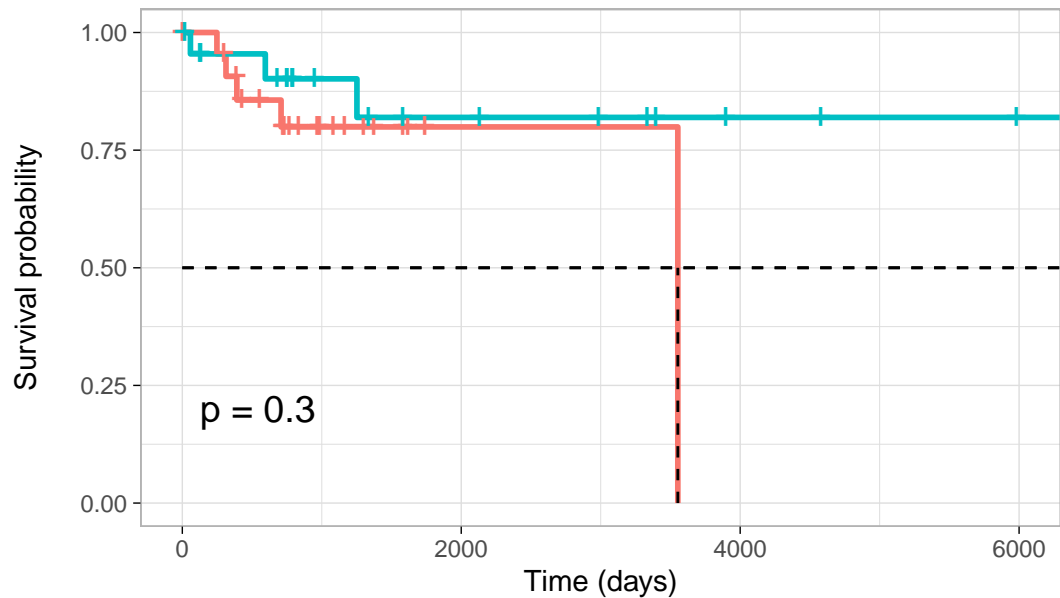

## Number at risk

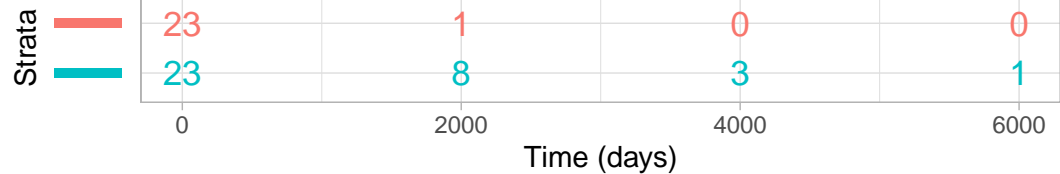

Supplement: S1 File — (ZIP) [file pone.0273163.s001.zip › Supplementary 1/5 UPF3B DLBC ╔·┤μ╖╓╬÷.pdf]

# ESCA UPF3B Survival

Strata + Gene=High expression (91) + Gene=Low expression (91)

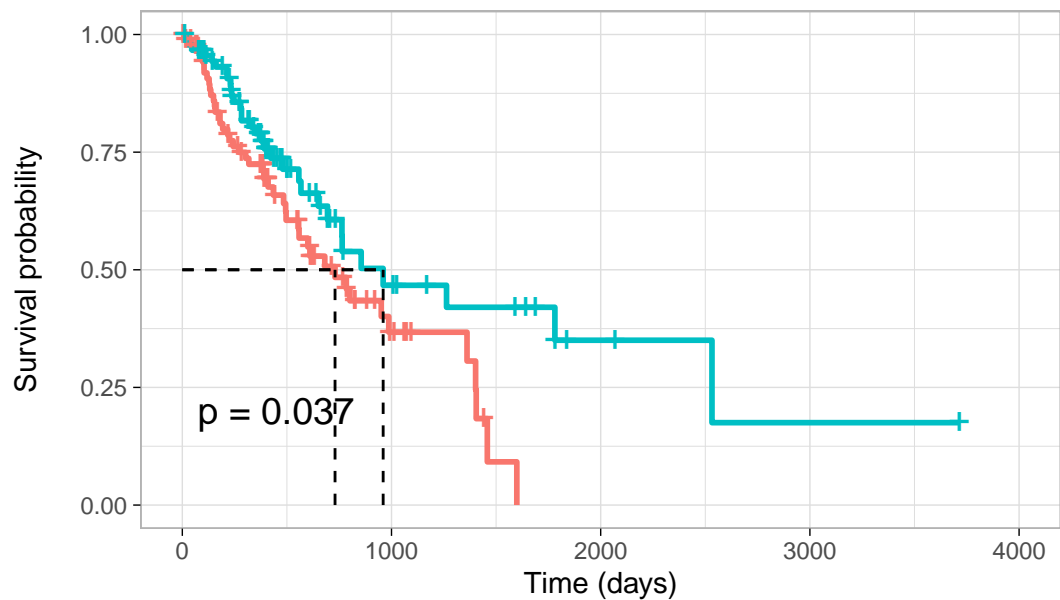

## Number at risk

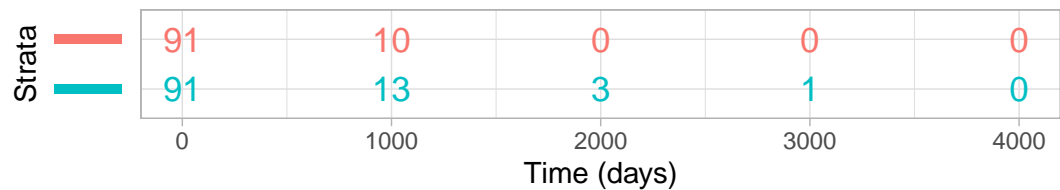

Supplement: S1 File — (ZIP) [file pone.0273163.s001.zip › Supplementary 1/5 UPF3B ESCA ╔·┤μ╖╓╬÷.pdf]

# GBM UPF3B Survival

Strata + Gene=High expression (83) + Gene=Low expression (82)

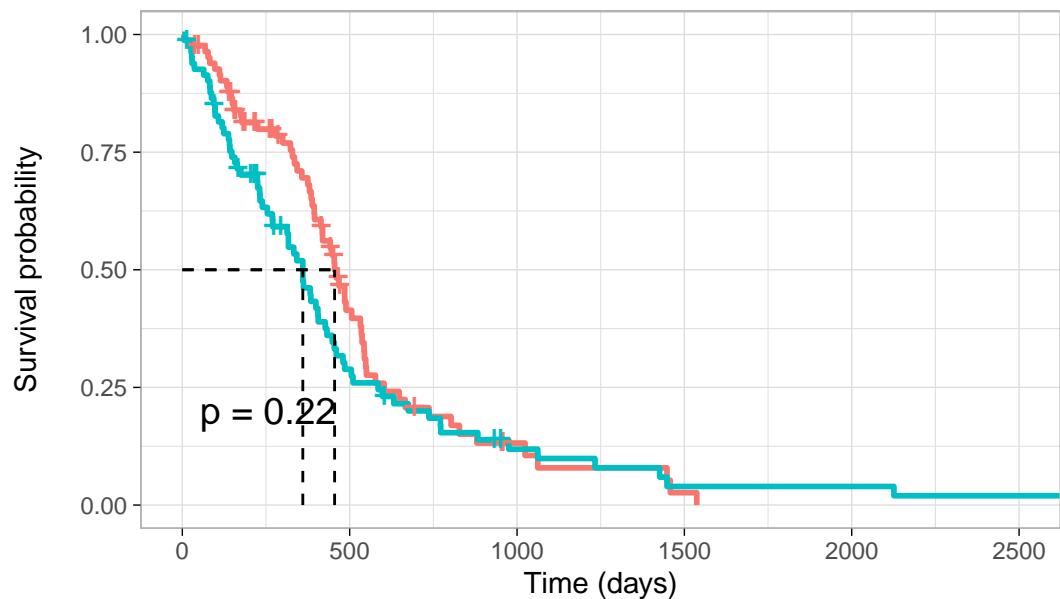

## Number at risk

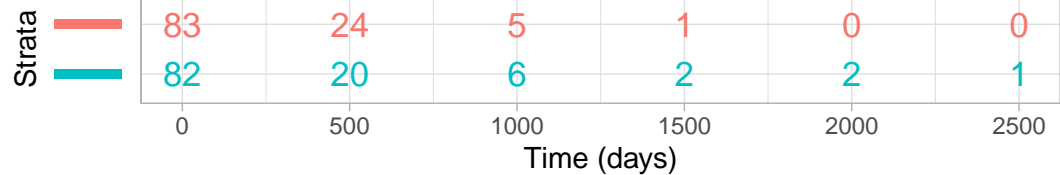

Supplement: S1 File — (ZIP) [file pone.0273163.s001.zip › Supplementary 1/5 UPF3B GBM ╔·┤μ╖╓╬÷.pdf]

# HNSC UPF3B Survival

Strata + Gene=High expression (260) + Gene=Low expression (259)

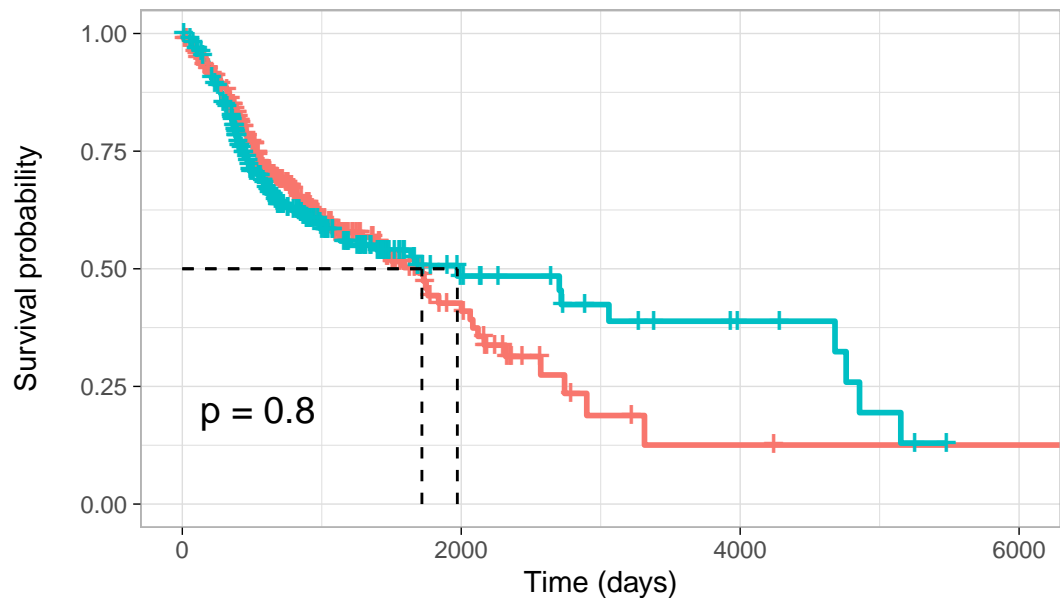

## Number at risk

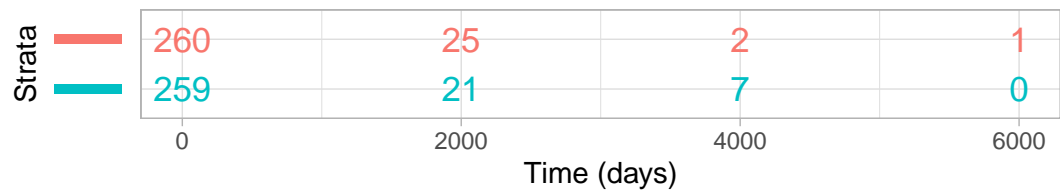

Supplement: S1 File — (ZIP) [file pone.0273163.s001.zip › Supplementary 1/5 UPF3B HNSC ╔·┤μ╖╓╬÷.pdf]

# KICH UPF3B Survival

Strata + Gene=High expression (33) + Gene=Low expression (32)

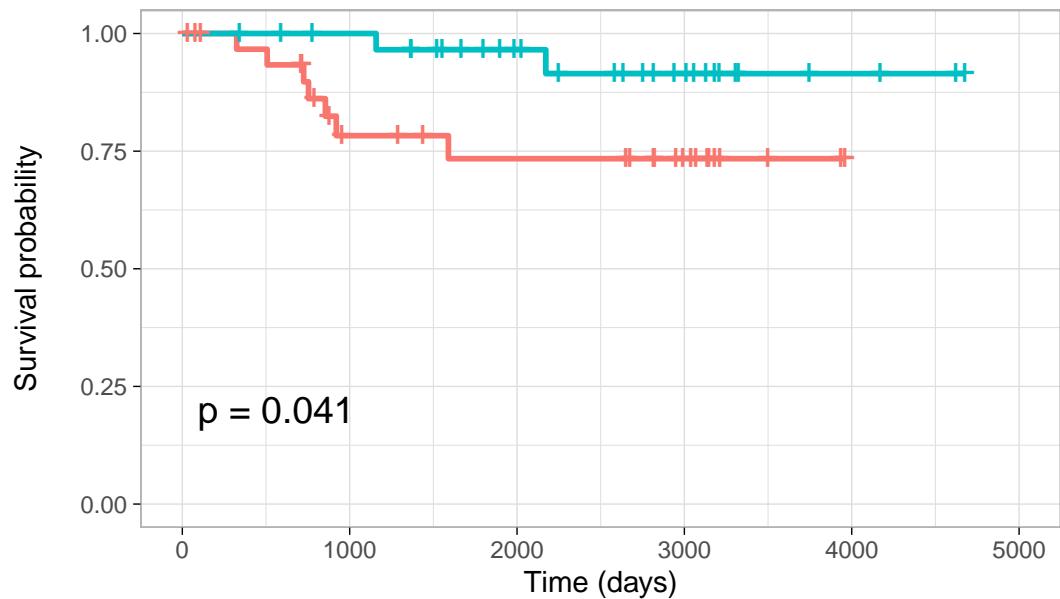

## Number at risk

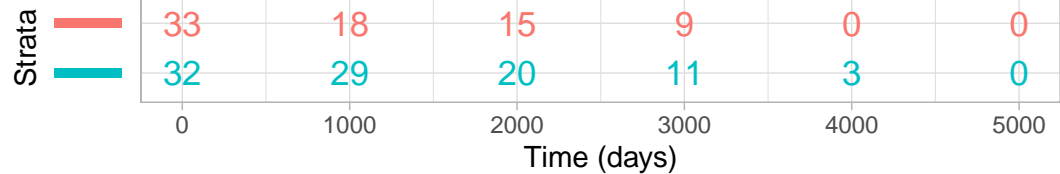

Supplement: S1 File — (ZIP) [file pone.0273163.s001.zip › Supplementary 1/5 UPF3B KICH ╔·┤μ╖╓╬÷.pdf]

# KIRC UPF3B Survival

Strata    + Gene=High expression (265)    + Gene=Low expression (264)

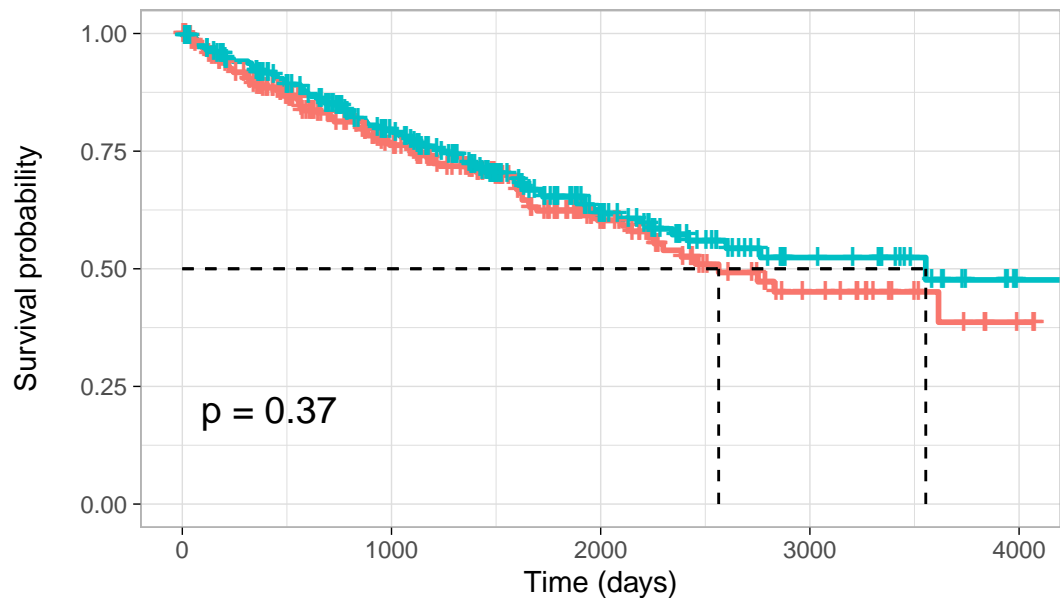

## Number at risk

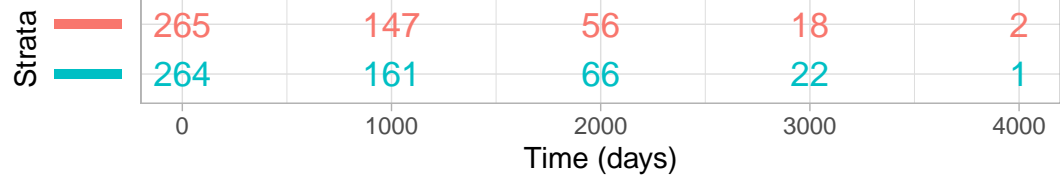

Supplement: S1 File — (ZIP) [file pone.0273163.s001.zip › Supplementary 1/5 UPF3B KIRC ╔·┤μ╖╓╬÷.pdf]

# KIRP UPF3B Survival

Strata    +    Gene=High expression (143)    +    Gene=Low expression (143)

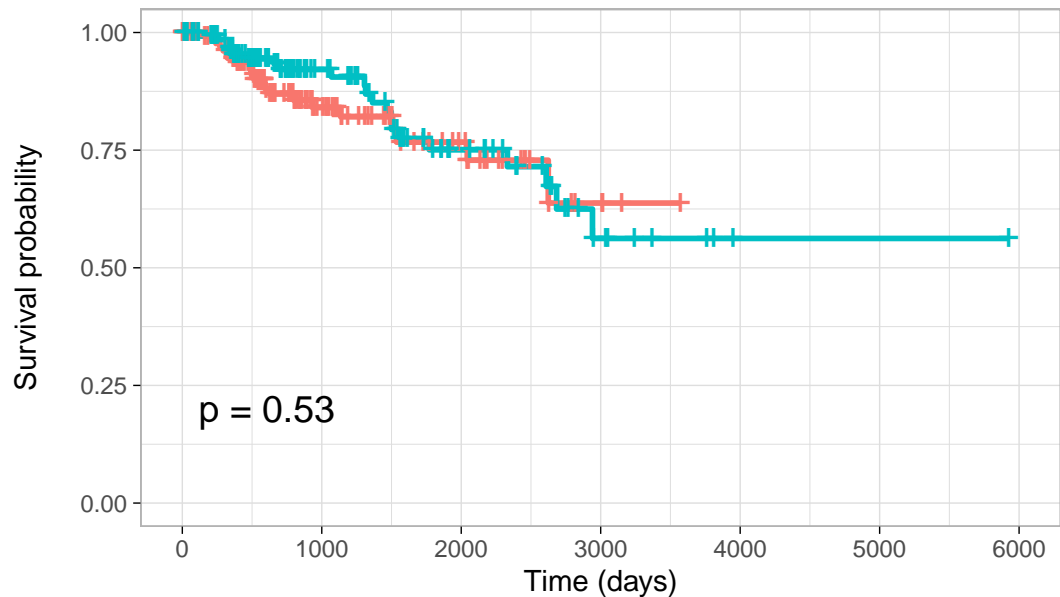

## Number at risk

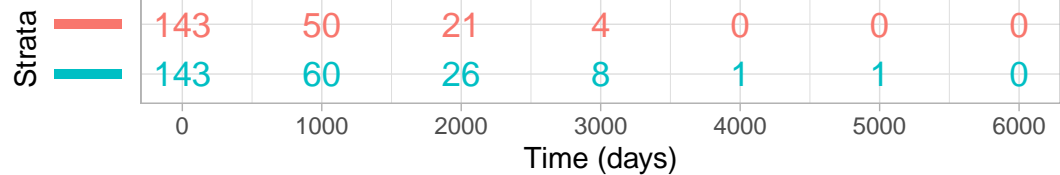

Supplement: S1 File — (ZIP) [file pone.0273163.s001.zip › Supplementary 1/5 UPF3B KIRP ╔·┤μ╖╓╬÷.pdf]

# LAML UPF3B Survival

Strata + Gene=High expression (75) + Gene=Low expression (74)

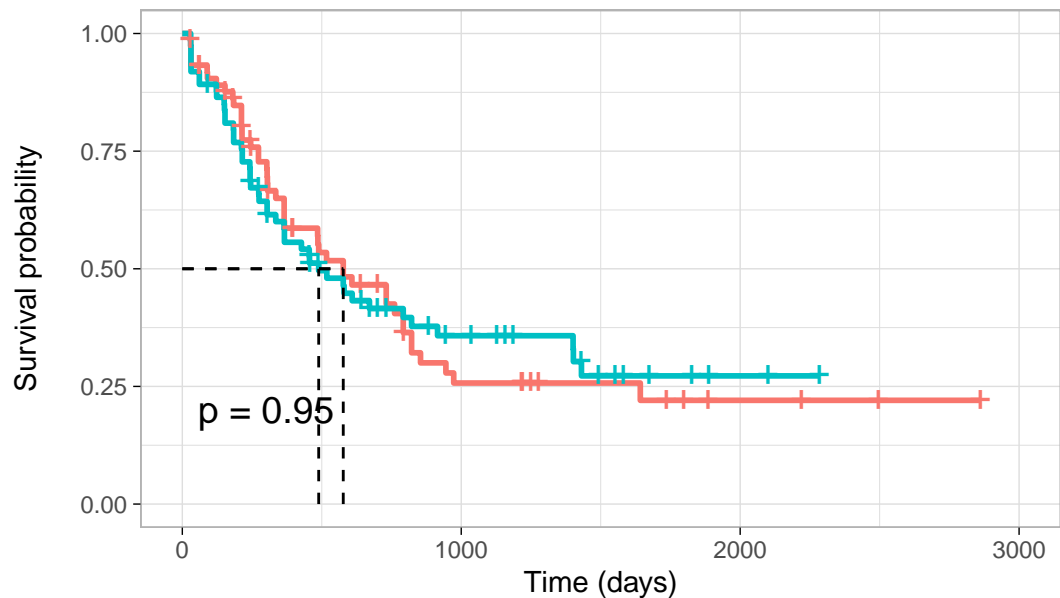

## Number at risk

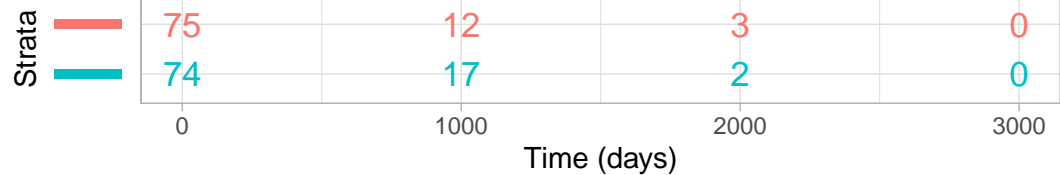

Supplement: S1 File — (ZIP) [file pone.0273163.s001.zip › Supplementary 1/5 UPF3B LAML ╔·┤μ╖╓╬÷.pdf]

# LGG UPF3B Survival

Strata + Gene=High expression (260) + Gene=Low expression (258)

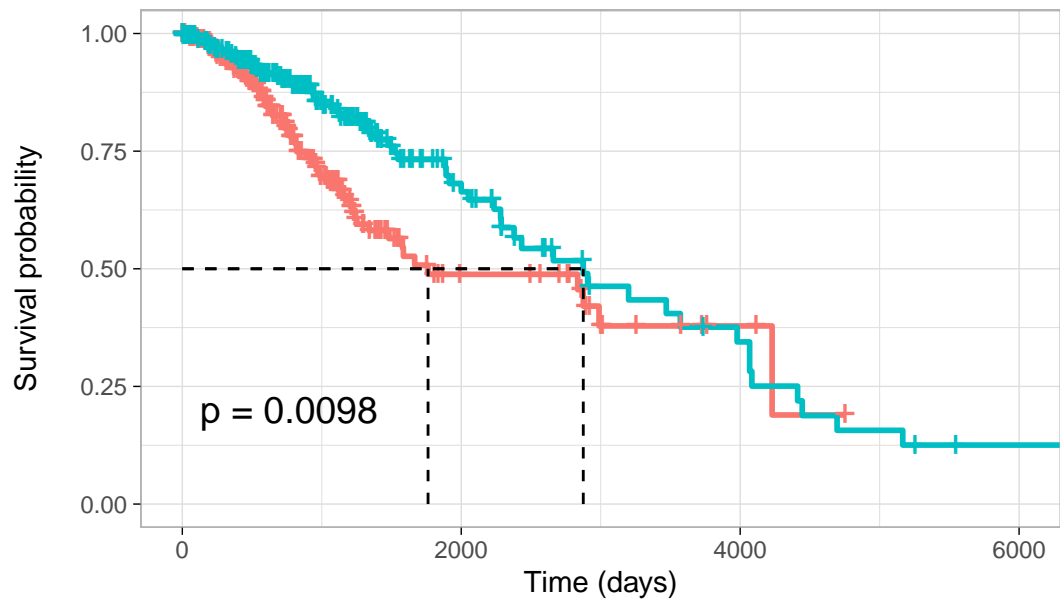

## Number at risk

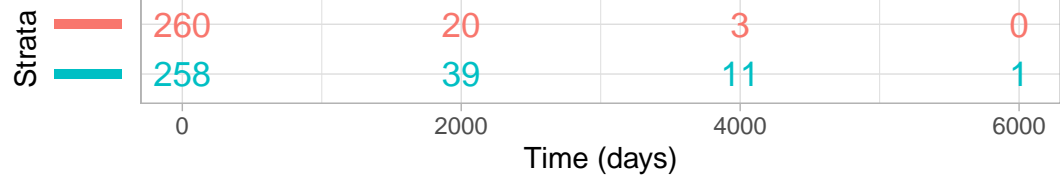

Supplement: S1 File — (ZIP) [file pone.0273163.s001.zip › Supplementary 1/5 UPF3B LGG ╔·┤μ╖╓╬÷.pdf]

# LIHC UPF3B Survival

Strata    +    Gene=High expression (183)    +    Gene=Low expression (182)

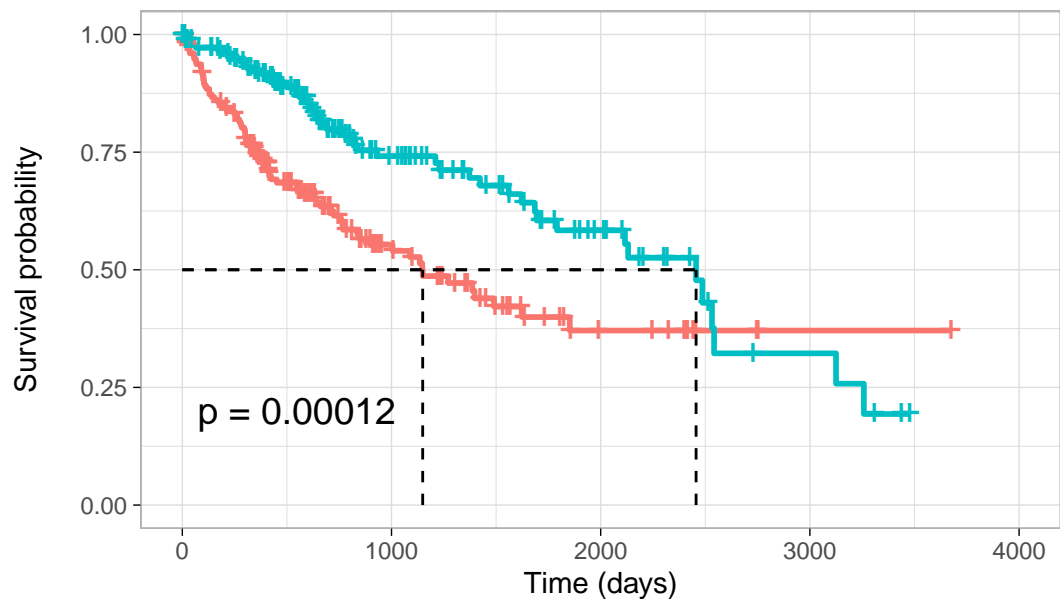

## Number at risk

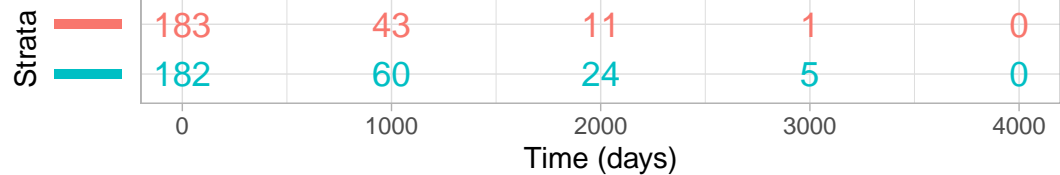

Supplement: S1 File — (ZIP) [file pone.0273163.s001.zip › Supplementary 1/5 UPF3B LIHC ╔·┤μ╖╓╬÷.pdf]

# LUAD UPF3B Survival

Strata + Gene=High expression (251) + Gene=Low expression (251)

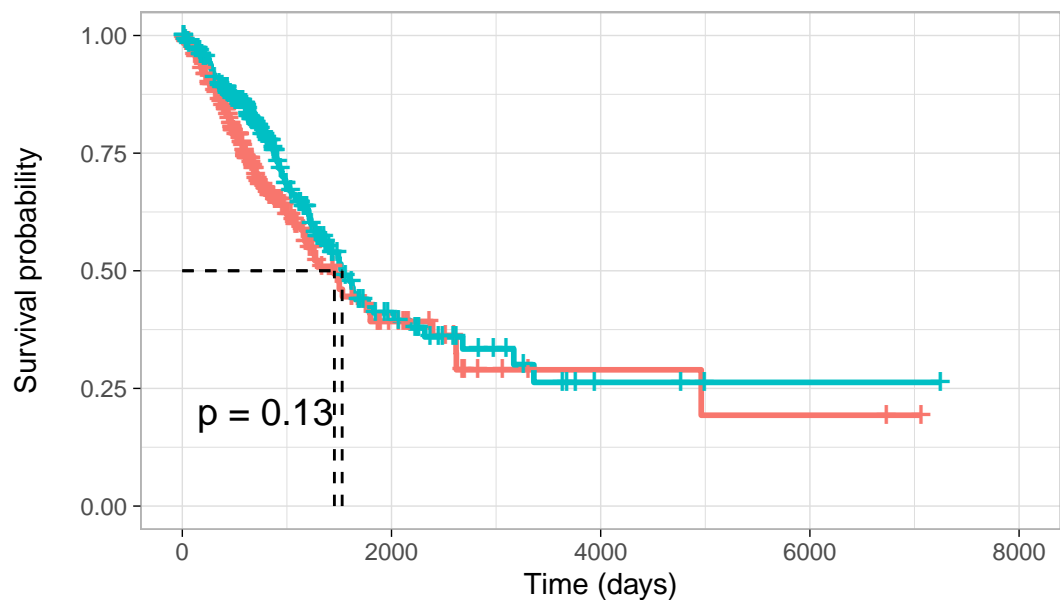

## Number at risk

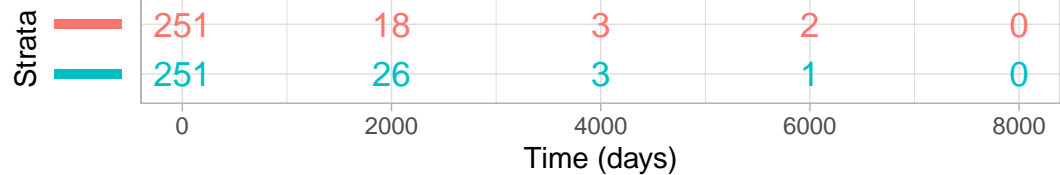

Supplement: S1 File — (ZIP) [file pone.0273163.s001.zip › Supplementary 1/5 UPF3B LUAD ╔·┤μ╖╓╬÷.pdf]

# LUSC UPF3B Survival

Strata + Gene=High expression (246) + Gene=Low expression (245)

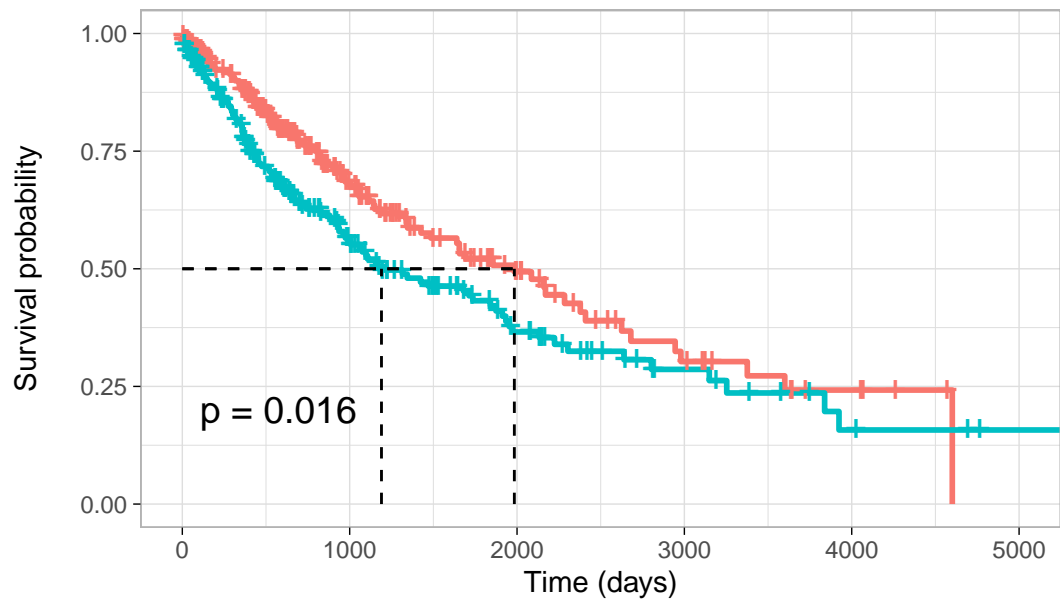

## Number at risk

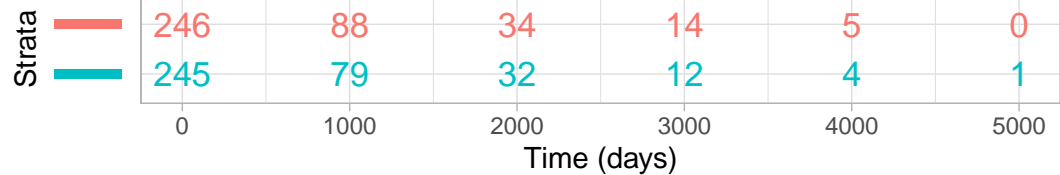

Supplement: S1 File — (ZIP) [file pone.0273163.s001.zip › Supplementary 1/5 UPF3B LUSC ╔·┤μ╖╓╬÷.pdf]

# MESO UPF3B Survival

Strata + Gene=High expression (43) + Gene=Low expression (42)

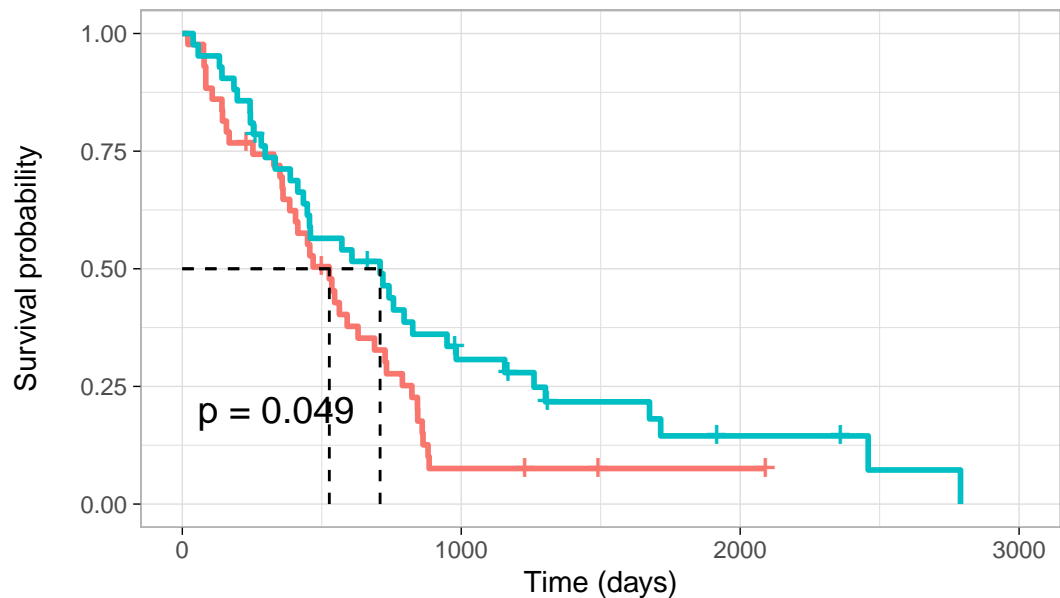

## Number at risk

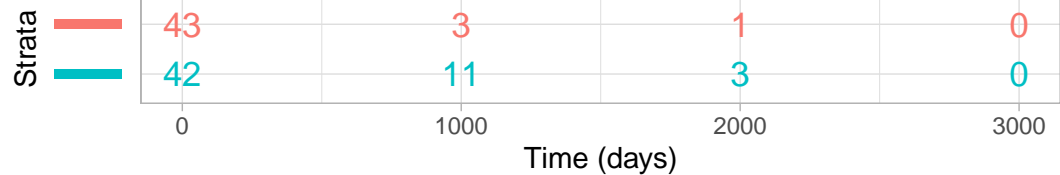

Supplement: S1 File — (ZIP) [file pone.0273163.s001.zip › Supplementary 1/5 UPF3B MESO ╔·┤μ╖╓╬÷.pdf]

# OV UPF3B Survival

Strata + Gene=High expression (213) + Gene=Low expression (212)

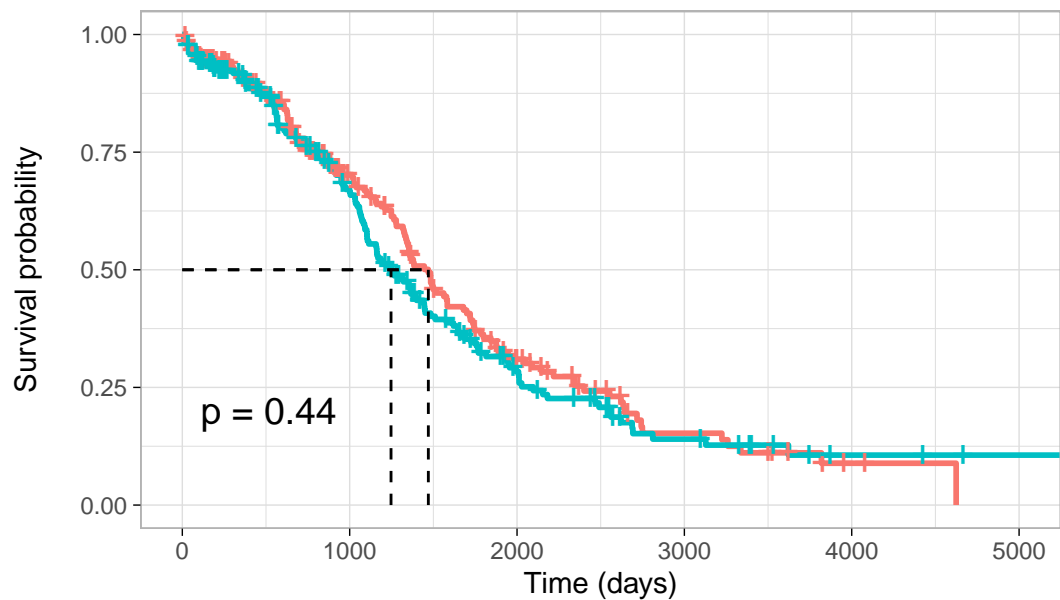

## Number at risk

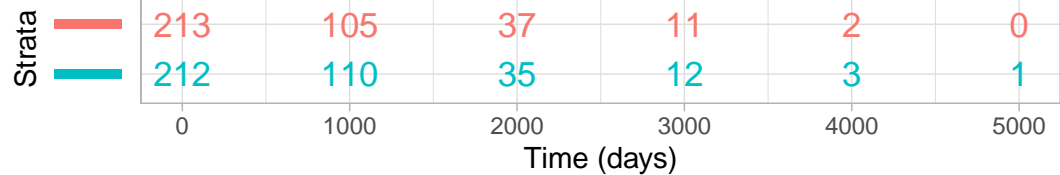

Supplement: S1 File — (ZIP) [file pone.0273163.s001.zip › Supplementary 1/5 UPF3B OV ╔·┤μ╖╓╬÷.pdf]

# PAAD UPF3B Survival

Strata + Gene=High expression (89) + Gene=Low expression (89)

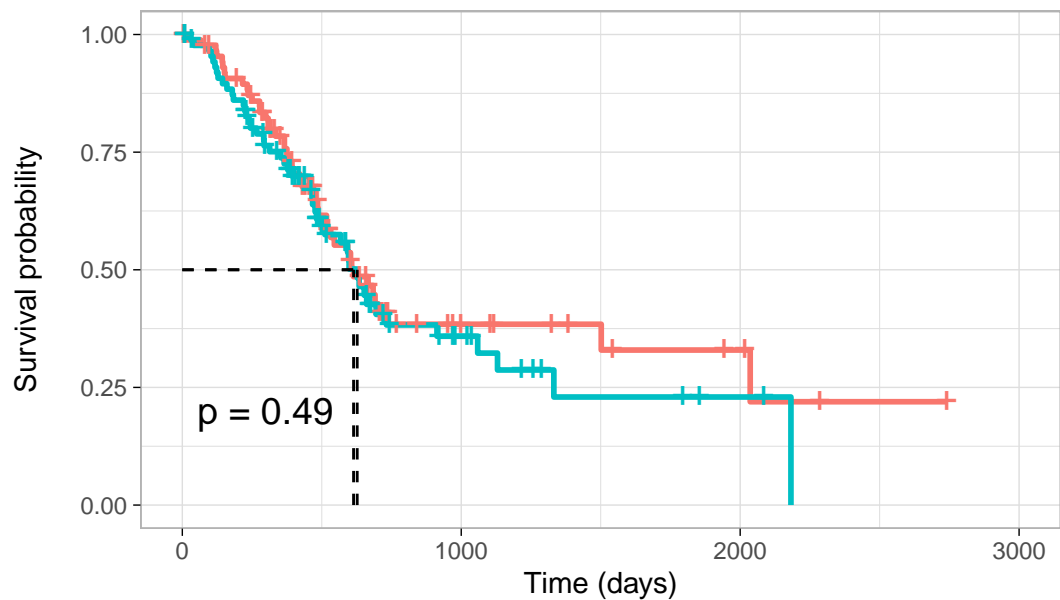

## Number at risk

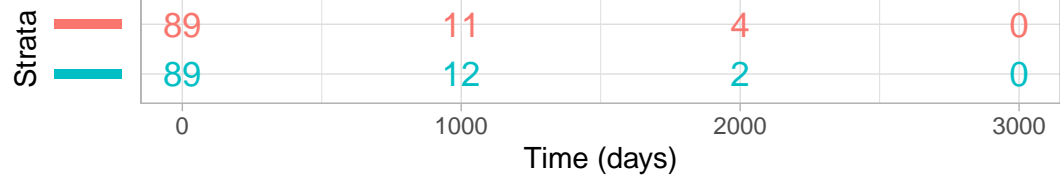

Supplement: S1 File — (ZIP) [file pone.0273163.s001.zip › Supplementary 1/5 UPF3B PAAD ╔·┤μ╖╓╬÷.pdf]

# PCPG UPF3B Survival

Strata + Gene=High expression (91) + Gene=Low expression (91)

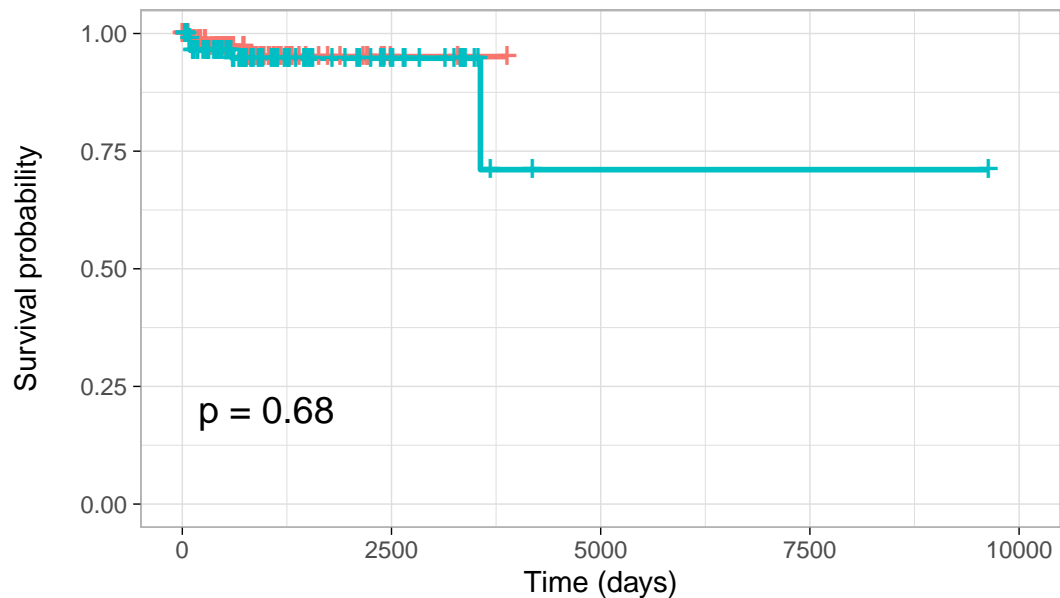

## Number at risk

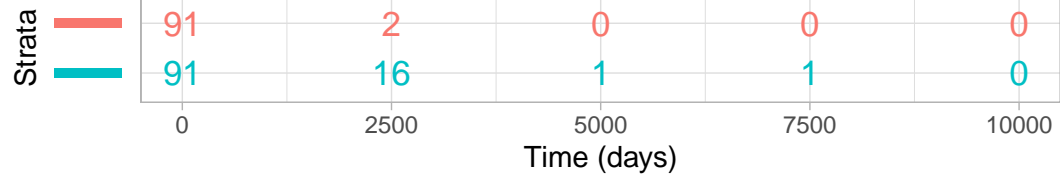

Supplement: S1 File — (ZIP) [file pone.0273163.s001.zip › Supplementary 1/5 UPF3B PCPG ╔·┤μ╖╓╬÷.pdf]

# PRAD UPF3B Survival

Strata + Gene=High expression (248) + Gene=Low expression (248)

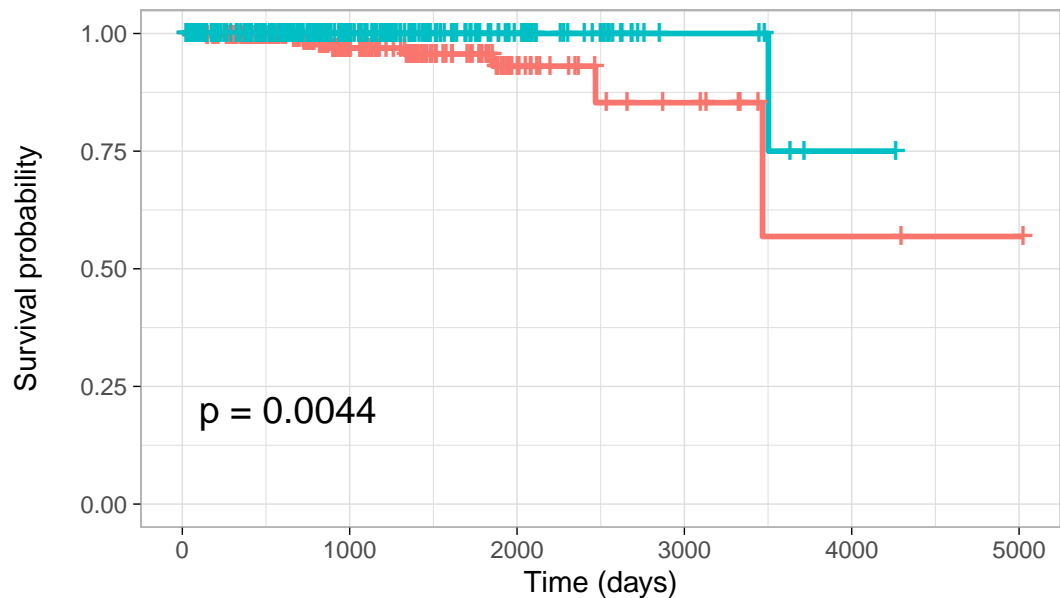

## Number at risk

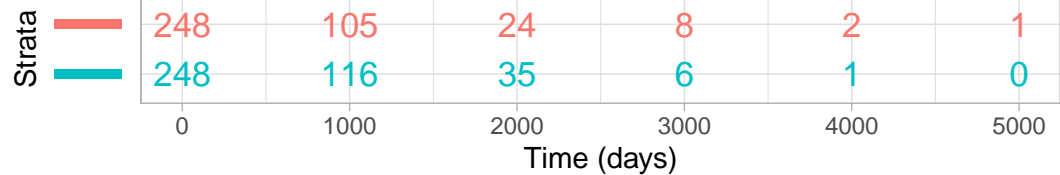

Supplement: S1 File — (ZIP) [file pone.0273163.s001.zip › Supplementary 1/5 UPF3B PRAD ╔·┤μ╖╓╬÷.pdf]

# READ UPF3B Survival

Strata + Gene=High expression (46) + Gene=Low expression (46)

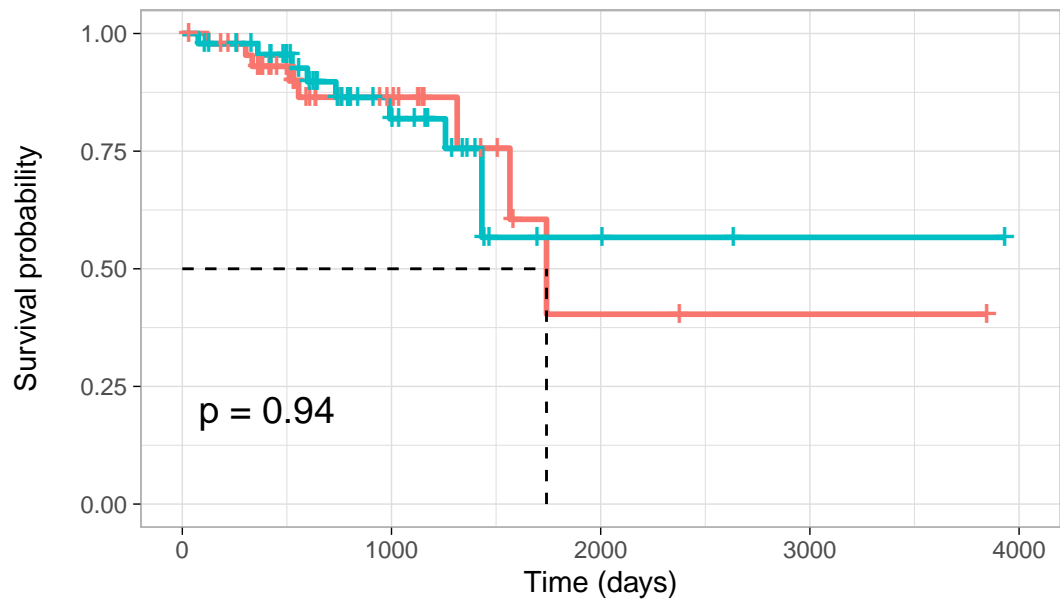

## Number at risk

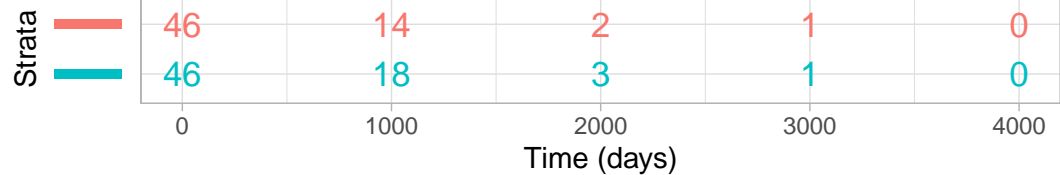

Supplement: S1 File — (ZIP) [file pone.0273163.s001.zip › Supplementary 1/5 UPF3B READ ╔·┤μ╖╓╬÷.pdf]

# SARC UPF3B Survival

Strata + Gene=High expression (131) + Gene=Low expression (131)

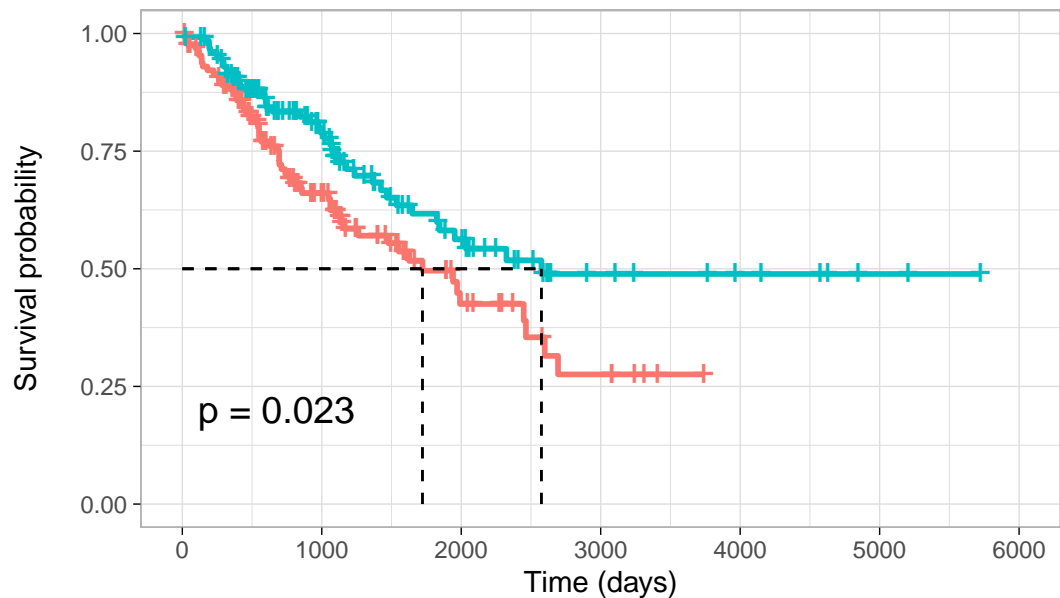

## Number at risk

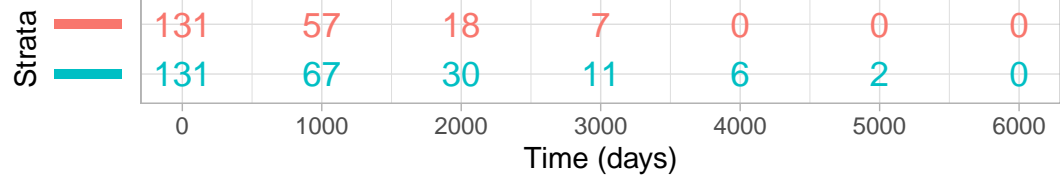

Supplement: S1 File — (ZIP) [file pone.0273163.s001.zip › Supplementary 1/5 UPF3B SARC ╔·┤μ╖╓╬÷.pdf]

# SKCM UPF3B Survival

Strata + Gene=High expression (227) + Gene=Low expression (226)

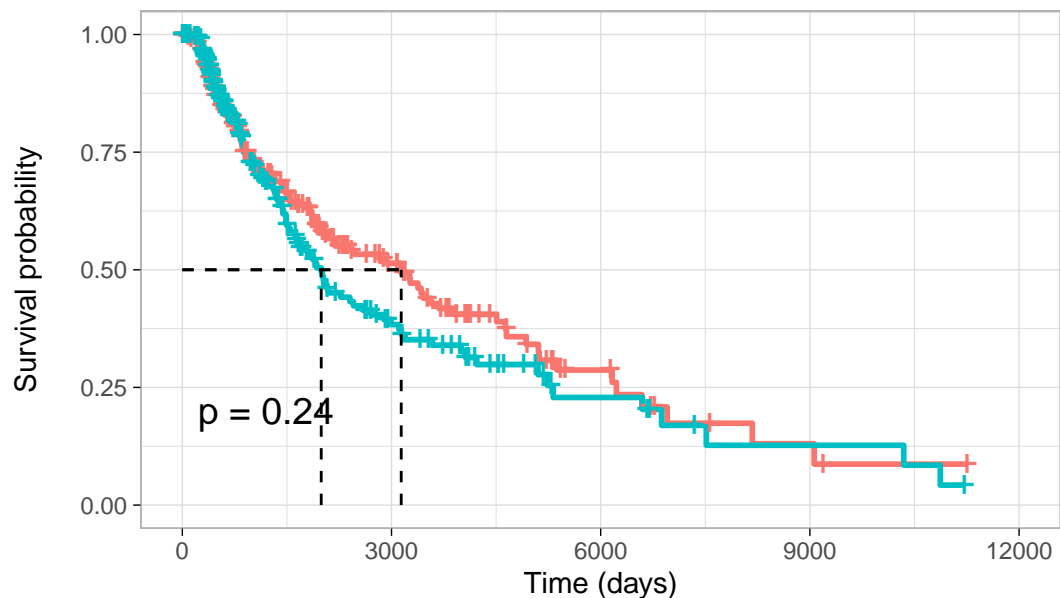

## Number at risk

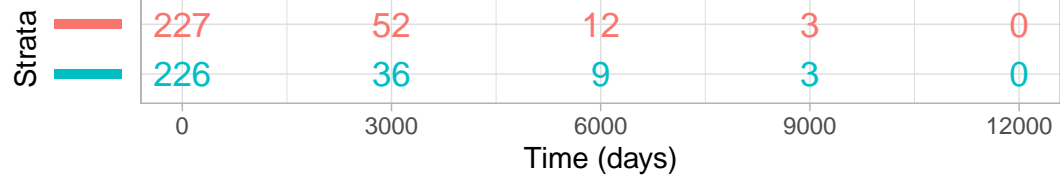

Supplement: S1 File — (ZIP) [file pone.0273163.s001.zip › Supplementary 1/5 UPF3B SKCM ╔·┤μ╖╓╬÷.pdf]

# STAD UPF3B Survival

Strata + Gene=High expression (194) + Gene=Low expression (194)

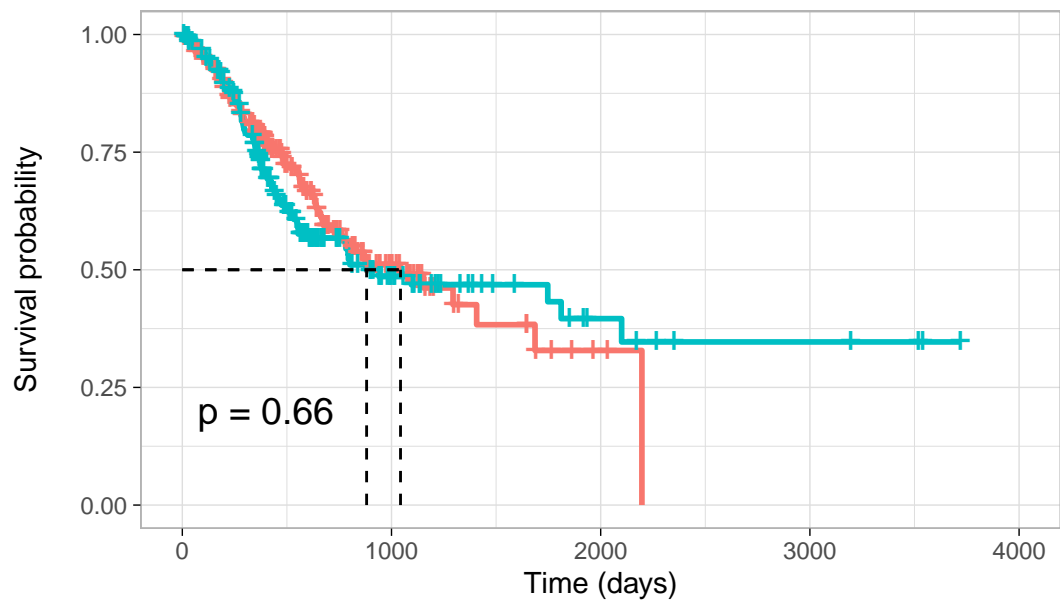

## Number at risk

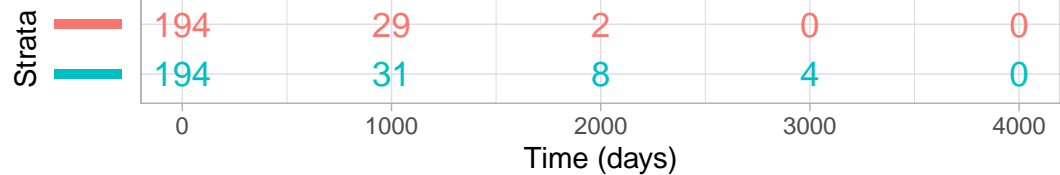

Supplement: S1 File — (ZIP) [file pone.0273163.s001.zip › Supplementary 1/5 UPF3B STAD ╔·┤μ╖╓╬÷.pdf]

# TGCT UPF3B Survival

Strata    +    Gene=High expression (69)    +    Gene=Low expression (68)

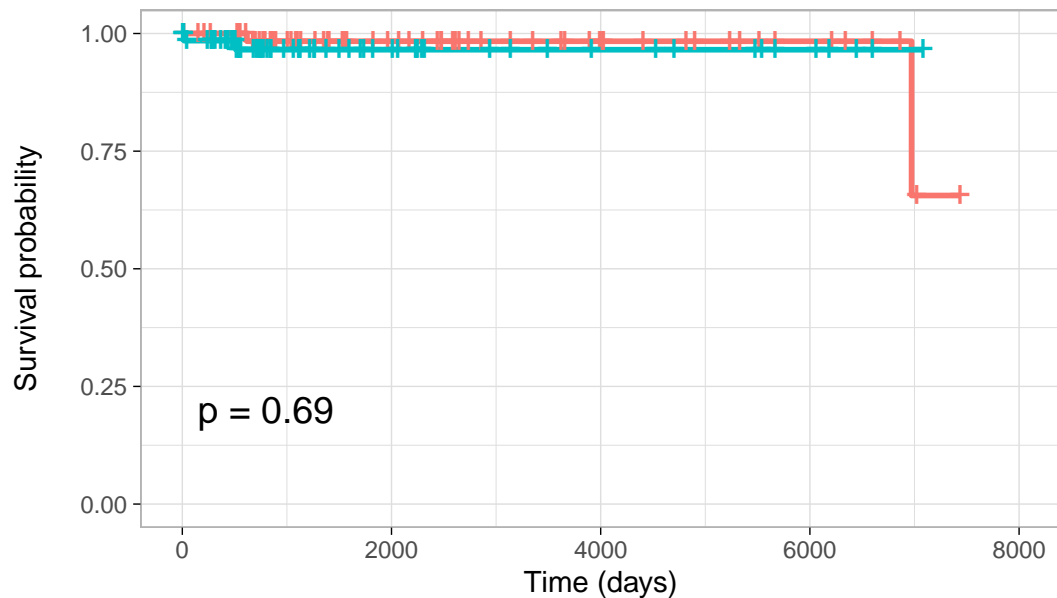

## Number at risk

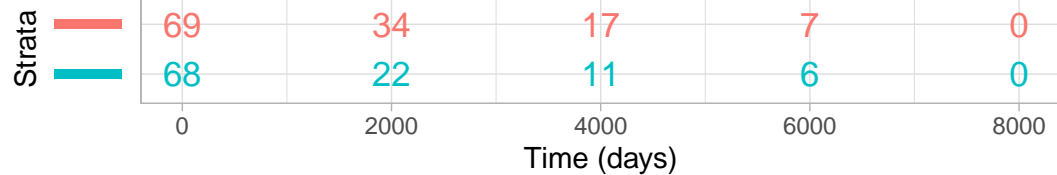

Supplement: S1 File — (ZIP) [file pone.0273163.s001.zip › Supplementary 1/5 UPF3B TGCT ╔·┤μ╖╓╬÷.pdf]

# THCA UPF3B Survival

Strata    +    Gene=High expression (258)    +    Gene=Low expression (253)

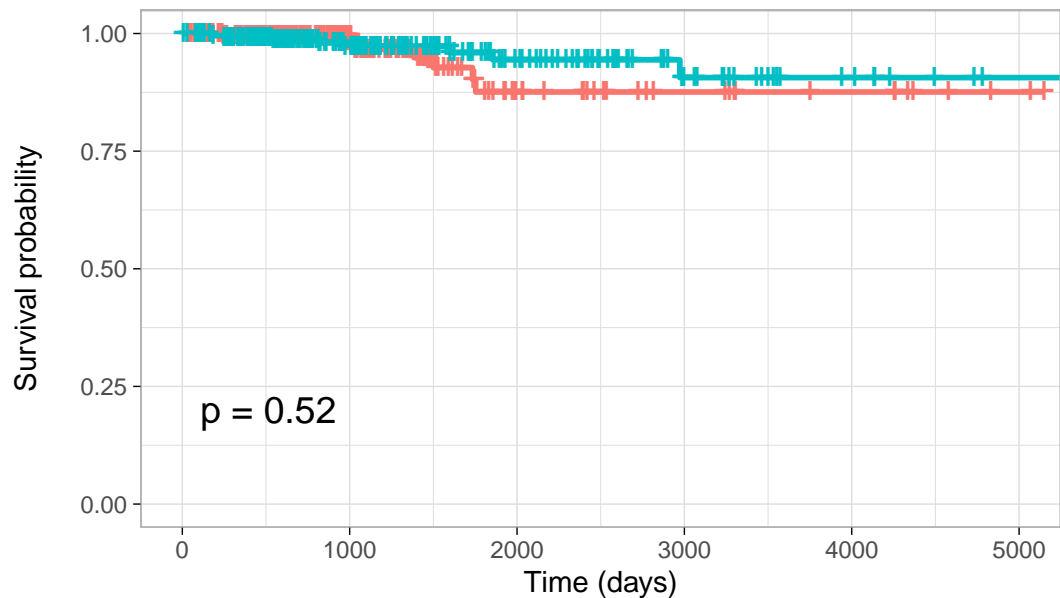

## Number at risk

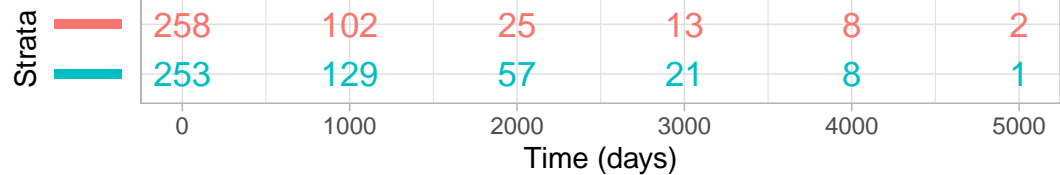

Supplement: S1 File — (ZIP) [file pone.0273163.s001.zip › Supplementary 1/5 UPF3B THCA ╔·┤μ╖╓╬÷.pdf]

# THYM UPF3B Survival

Strata + Gene=High expression (59) + Gene=Low expression (59)

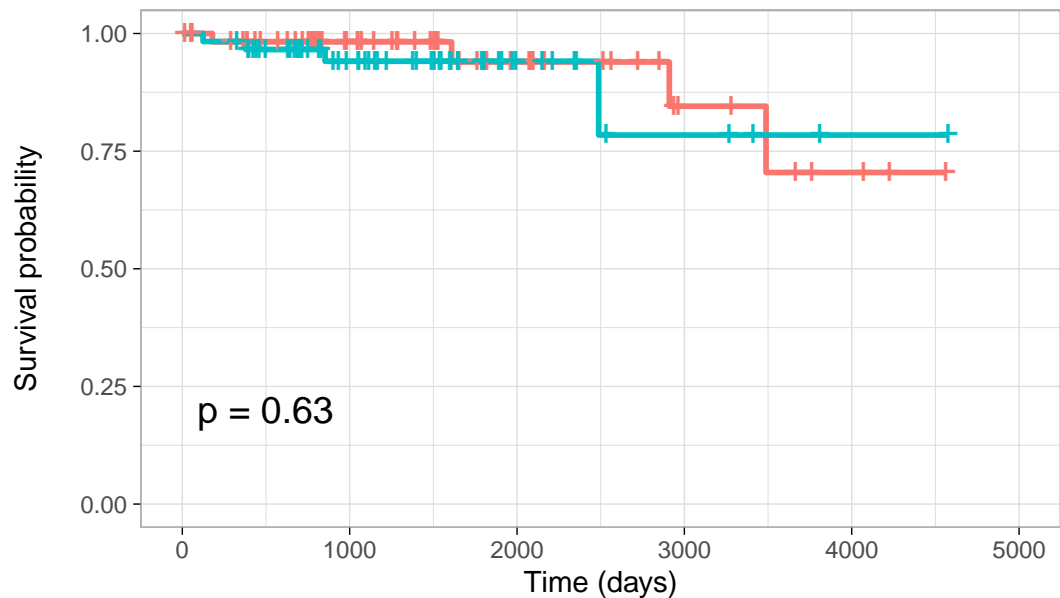

## Number at risk

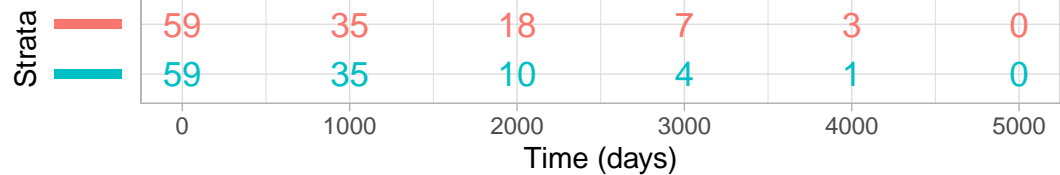

Supplement: S1 File — (ZIP) [file pone.0273163.s001.zip › Supplementary 1/5 UPF3B THYM ╔·┤μ╖╓╬÷.pdf]

# UCEC UPF3B Survival

Strata    +    Gene=High expression (90)    +    Gene=Low expression (89)

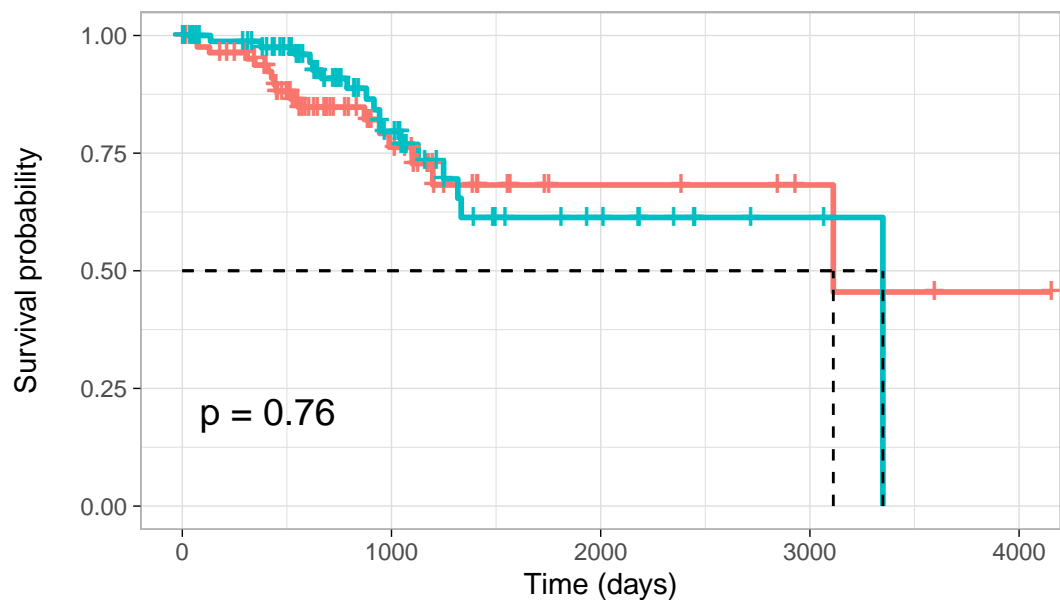

## Number at risk

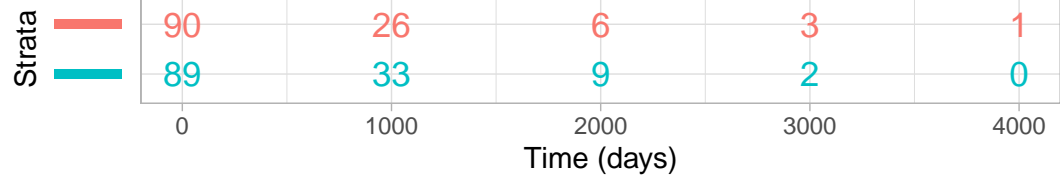

Supplement: S1 File — (ZIP) [file pone.0273163.s001.zip › Supplementary 1/5 UPF3B UCEC ╔·┤μ╖╓╬÷.pdf]

# UCS UPF3B Survival

Strata + Gene=High expression (28) + Gene=Low expression (28)

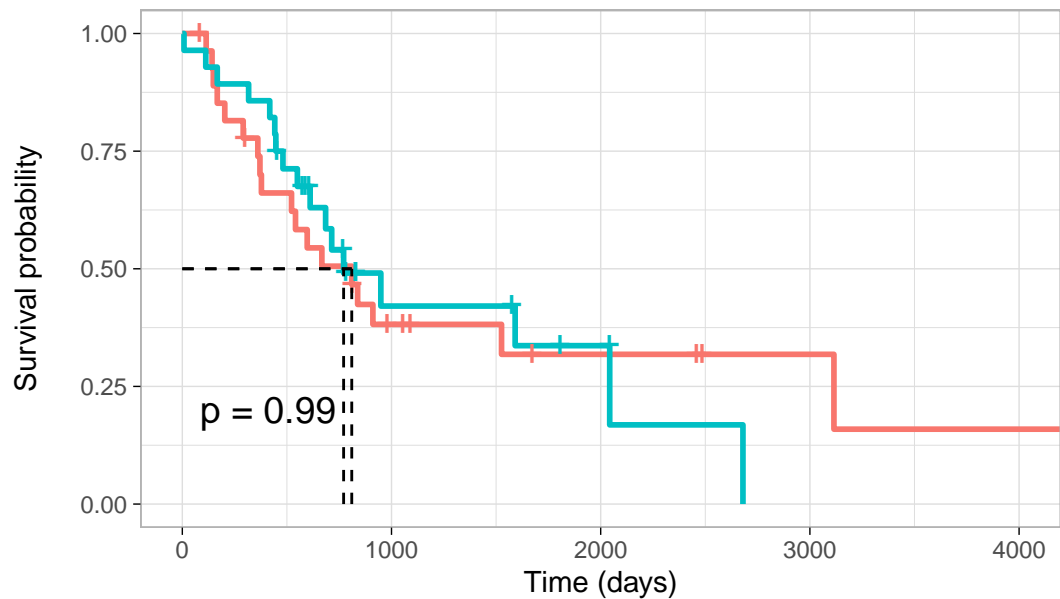

## Number at risk

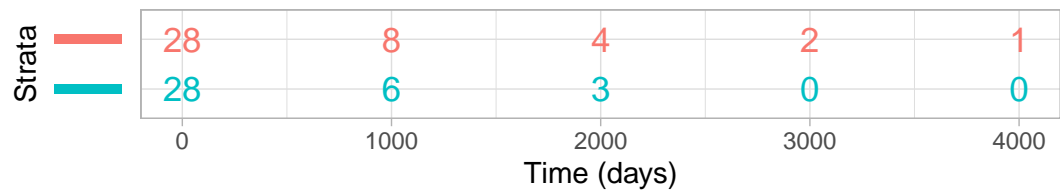

Supplement: S1 File — (ZIP) [file pone.0273163.s001.zip › Supplementary 1/5 UPF3B UCS ╔·┤μ╖╓╬÷.pdf]

# UVM UPF3B Survival

Strata + Gene=High expression (40) + Gene=Low expression (39)

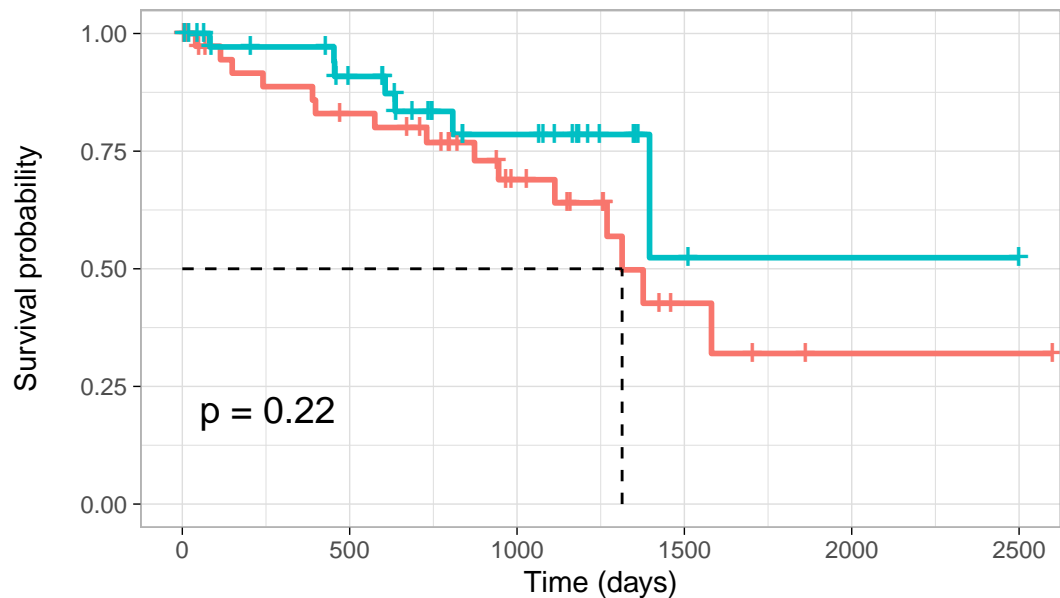

## Number at risk

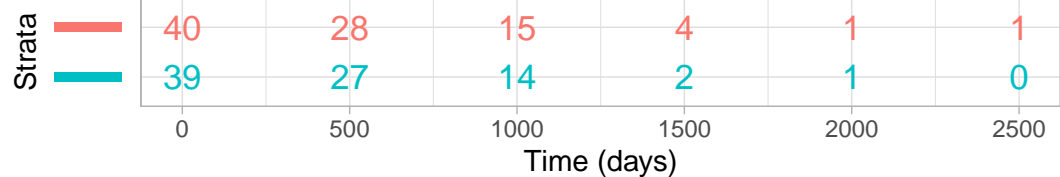

Supplement: S1 File — (ZIP) [file pone.0273163.s001.zip › Supplementary 1/5 UPF3B UVM ╔·┤μ╖╓╬÷.pdf]
